# Insight into the Mechanisms of Nitride Films with Excellent Hardness and Lubricating Performance: A Review

**DOI:** 10.3390/nano13152205

**Published:** 2023-07-29

**Authors:** Xinmeng Wu, Yaohong Jiang, Tianhao Wu, Bin Zuo, Shunuo Bian, Kun Lu, Lijun Zhao, Lihua Yu, Junhua Xu

**Affiliations:** 1 Department of Material and Science, Jiangsu University of Science and Technology, Zhenjiang 212000, China; 2 Department of Medicine, Chuzhou City Vocational College, Chuzhou 239000, China

**Keywords:** nitride composite films, design strategy, strengthening mechanisms, lubricating mechanisms

## Abstract

Transition metal nitride (TMN) films with excellent hardness and lubricating performance are versatile low dimension materials, which are widely used in various fields including industries, transportation, aerospace, and so on. This paper introduces one film design strategy and provides a review of the mechanisms for strengthening and lubricating nitride films. The design strategy refers to two aspects which determine the structures, the performance, the components, and the chemical constitutions of nitride films The strengthening mechanisms of nitride films are then illuminated in detail, including the solid solution effect, the grain size effect, the secondary phase effect, the stress or stress field effect, the template effect, and the valence electron concentration effect. Five lubricating mechanisms are next summarized, including the easy-shear nature, the tribo-chemical reactions, the lubricious fluorides, the textured contact surface, and the synergistic effect. This paper aims to give a comprehensive introduction for understanding the mechanisms of strengthening and lubrication of nitride films for students and researchers, as well as to understand the current research progress in nitride films for exploring research gaps.

## 1. Introduction

**Figure 1:**
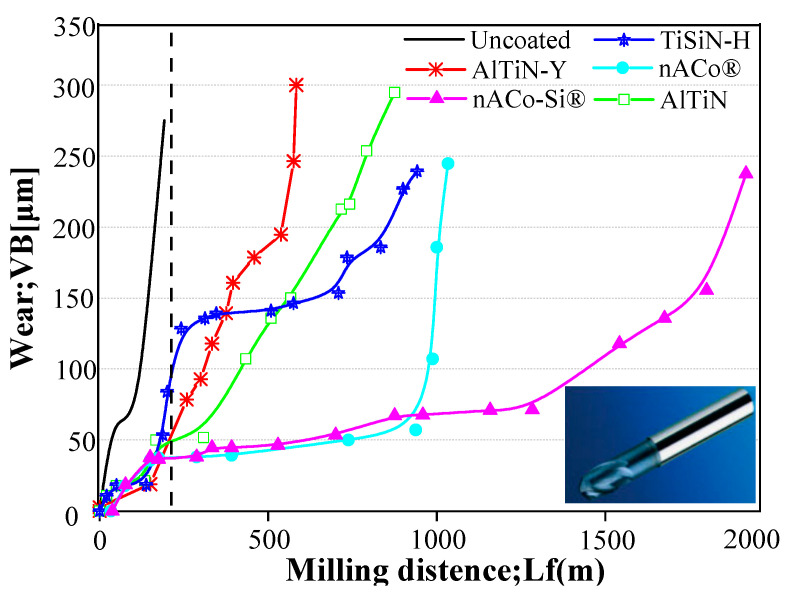
Milling distance of 56 HRC hard steel made of cemented carbide coated with different coatings.

Studying both friction and wear is the main task of our current research, as friction consumes more than 30% of the world’s primary energy [1,2], and the wear between moving parts is one of the main causes of material and mechanical equipment failure [3]. In the last decades, science researchers have spared no effort to lower the friction coefficient and wear rate by surface functionality, which is one of the most effective methods. This is much more meaningful for addressing the energy shortage problem all over the world, as it raises the efficiency of energy utilization through decreasing friction and wear. Vapor deposition is known as a superb surface engineering technology that can meet extreme requirements. It is commonly suggested to prepare nitride films with premium hardness, even superhardness, and excellent lubrication performance on the cutting tools [4]. On the other hand, PVD is widely recommended for its green development and high efficiency. Compared to uncoated coatings, PVD coatings can improve the surface quality of workpieces and extend the life of tools [5], as shown in Figure 1. Conventional binary nitride films [6,7,8,9], such as TiN/CrN/ZrN, are widely used due to their chemical stability and mechanical properties, etc., but they do not satisfy the hardness and lubrication demands that are expected in some fields. When it comes to the lubricating contributions of TiN [10], CrN [11], ZrN [12], and other nitriding films in the field of tribology, they are absolutely satisfactory as classic Generation-I films. However, modern manufacturing industries require more stringent requirements, such as setting extremely high surface finish levels. Since the 1990s, myriad reports have claimed that the addition of silicon [13] and/or aluminum [14] to films plays a remarkable role in improving their mechanical and tribological properties. These films are generally classified as Generation-II according to C. Donnet and Erdemir [15].

In 1974, Norio Taniguchi, a Japanese scientist from Tokyo University of Science, first proposed the concept of nanometers and coined the term “nano-technology” as “The processing of separation, consolidation, and deformation of materials by one atom or one molecule” [16]. Since then, nanotechnology has come into existence, and based on it, different kinds of nano-films and composite films have been developed [17,18,19,20,21]. Some key advantages of nano-structured and composite coatings over traditional coatings are their superior mechanical hardness [22], resilience [23], toughness [24], high resistance to wear [25], scuffing [26], fatigue [27], oxidation [28], corrosion [29], lower friction [30,31], and ability to produce low shear tribo-films on dry or marginally lubricated sliding surfaces with self-adaptive behavior in variable environments and temperatures [32,33].

**Figure 2:**
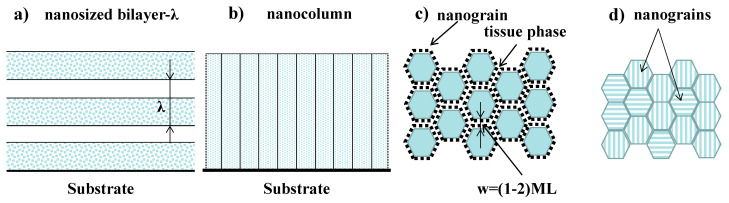
Schematic illustration of four classic nanostructures of the Generation-III coating, (**a**,**b**) are the 2D structures, (**c**,**d**) are the 3D structures, adapted from ref. [38].

**Figure 3:**
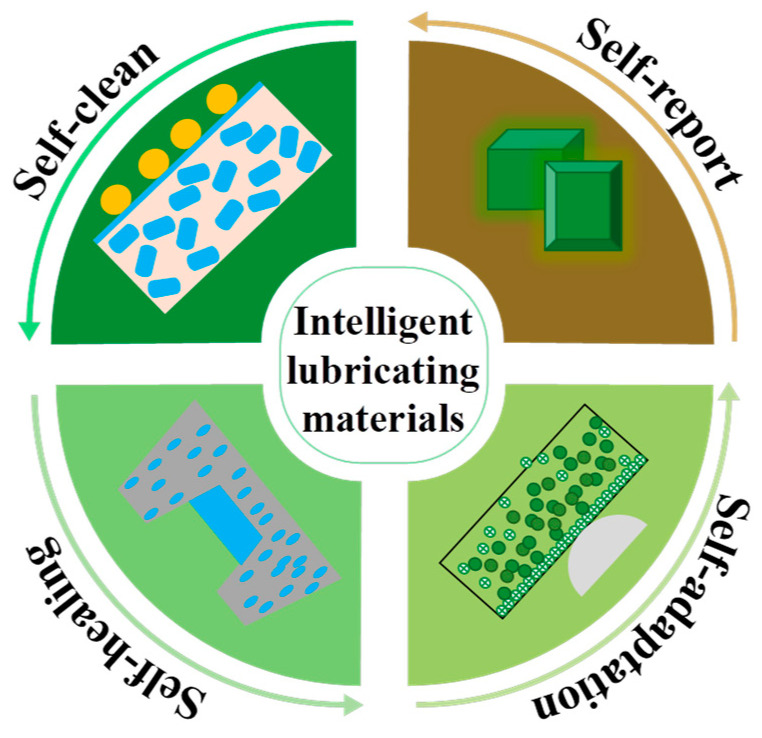
An illustration of four classic smart coatings.

Figure 2 gives a schematic illustration of the Generation-III films, including nano-size-bilayer structured ones, nano-column structured ones, nano-composite structured ones, and nano-grain structured ones. Absolutely, the smart nitride films provide a significant advantage over conventional films. Hanjun Gong et al. [34] declared the smart lubricating materials to be of four types, as shown in Figure 3: self-healing, self-clean, self-adaptation, and self-report, indicating a tendency in the surface engineering field. Some published works have reported smart coatings as chameleon coatings [35,36] based on their self-adaptation behaviors, which give satisfactory advantages (both mechanical and lubricating properties) over a wide temperature range [37].

**Figure 4:**
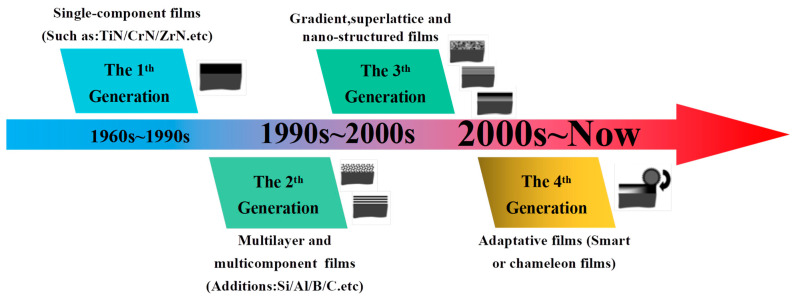
Historical developments of various nitride films.

Donnet and Erdemir et al. [15] classified the coatings into four generations: (1) the single-component coating, (2) the multilayered and multicomponent coating, (3) the gradient, superlattice, and nano-structured coating, and (4) the smart coating, as shown in Figure 4, which summarizes the historical development of protective and solid lubricant films. Up to now, various articles have been reported which mainly focus on their specific research works, and there have been few reviews focused on the strengthening and lubricating mechanisms of nitride films. The goal of this review is to give an overview of these mechanisms, which are responsible for the satisfactory hardness and excellent lubricating behavior. Here, we organize the article as follows: (1) First, give an introduction about one film design strategy and the influencing factors; (2) Second, elaborate the strengthening mechanisms; (3) Third, illuminate the lubricating mechanisms; (4) Finally, give a brief prospective for the future.

## 2. Design Considerations

Here, we will introduce one design strategy for fabricating nitride films with excellent hardness and lubricating performance. We will introduce this design strategy in two parts: (1) the film components; (2) the chemical constitutions. Since the film lubrication theory [39] was proposed, various films have been prepared to reduce the friction coefficient and wear rate, and the hardness and lubricating behavior of films are the main factors. Relations between hardness and wear models [40], as well as functional models [41], have been proposed as a purposeful and simple way to predict wear under those identified conditions. Evans and Marshall [42] were the first to estimate the wear of engineering ceramics with a lateral crack model in the case of a ball on a flat test configuration using a counterpart of a sharp radius. 

Estimating the volume of wear according to mechanical characteristics plays a definitive role in analyzing the wear process of brittle ceramics, and the relationship between the fracture toughness and hardness of materials was taken into consideration (Equation (1)).

(1) $$v=\alpha \cdot \frac{{F_{N}}^{9/8}}{K_{IC}^{1/2}\cdot H^{5/8}}\cdot {\left(\frac{E}{H}\right)}^{4/5}\cdot S$$

where v is wear volume; α is a material independent constant; F_N_ is the load (N); K_IC_ is the fracture toughness; H is the hardness (GPa); E is the elastic modulus (GPa); S is the total sliding distance (m).

**Figure 5:**
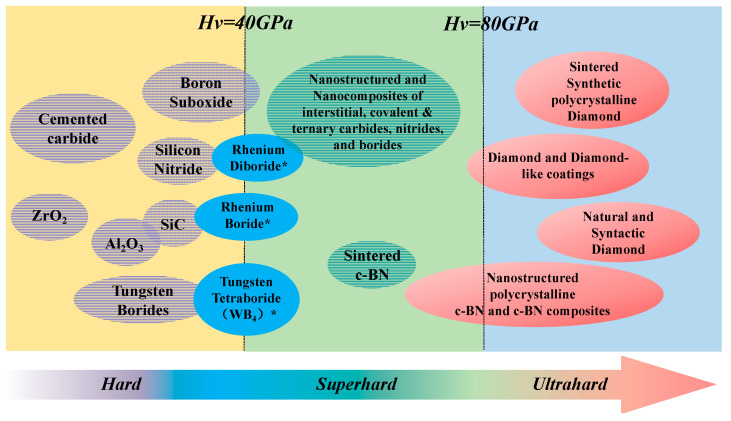
A schematic illustration of materials with various hardness. “*” means the hardness of the corresponding materials is in the transition region between hard and superhard.

Hardness is defined as the ability of materials to resist being scratched or dented by another material [43]. According to the hardness (H), the coatings are divided into three groups, as shown in Figure 5: (1) Hard coatings with H < 40 GPa; (2) Superhard coatings with H ranging from ~40 to ~80 GPa; (3) Ultrahard coatings with H > 80 GPa. It has been found that there are two groups of superhard nano-composite coatings [38,44,45]:

1. The two-phase formation model called hard phase/hard phase composite films, e.g., nc-Me1N/a-Me2N, etc. We denote the phase constitution model of hard/hard nanocomposites as Model I.
2. The formation model of hard phase/soft phase composite films is referred to as Model II, e.g., nc-Me1N/Me3, etc.

Here, Me1 = Ti, Zr, W, Ta, Cr, Mo, Al, etc., are strong nitride forming elements, Me2 = Si, B, C, etc., are p-elements, and Me3 = Cu, Ni, Ag, Au, Y, etc., are common metal elements used in Model II. Within, “nc” is short for nanocrystalline and “a” is short for amorphous. Model I and Model II demonstrate the potential to combine the distinct properties of these components in order to tailor the performance of composite films [15,38,45,46,47,48]. We will summarize relevant mechanisms later in this paper.

### 2.1. The Components

By synergistically considering the film components and chemical composition, it is possible to successfully prepare nitride films with excellent hardness and lubricating performance. Voevodin and coworkers [48] achieved the preparation of films with super hardness and excellent lubricating performance using a multi-component strategy of “hard matrix + lubricants + reinforcements”. This approach involved incorporating the amorphous phase as the matrix, utilizing crystalline and/or nanocrystalline solid lubricants as the lubricating component, and introducing encapsulated hard components as reinforcements. This type of nitride film is specifically composed of reinforcements and composite solid lubricants embedded in an amorphous matrix [3,49,50,51,52]. In the following sections, we will delve into a detailed discussion of the film components, including amorphous and/or nano/normal grain-sized crystal structures, which serve as the matrix [3,53,54], the reinforcements, and the lubricants, respectively. The phase chemical constitutions will be explored in Section 2.2. It is important to clarify that the amorphous phase referred to here is the X-ray amorphous phase [18].

#### 2.1.1. The Amorphous Matrixes

The matrix component plays a crucial role in providing structural support to the films under contact load and ensuring their integrity during friction. Some researchers [3] have emphasized that, as the matrix of nitride films, the amorphous phase effectively coordinates plastic deformation and mitigates stress, thereby enhancing film performance. The amorphous matrix can also impede nano-cracks by deflecting them at grain boundaries and dissipating energy. Additionally, the presence of amorphous phases prevents direct contact between the matrix, substrate, and corrosive environments [38], leading to improved oxidation resistance. J. Musil et al. [55] have reported that X-ray amorphous coatings exhibited outstanding oxidation resistance, even exceeding ~1000 °C. This remarkable performance can be attributed to the smaller grain size of the amorphous coatings compared to regular nanocrystals, which eliminates direct contact between the external atmosphere at the coating surface and the substrate surface via grain boundaries [38]. 

**Figure 6:**
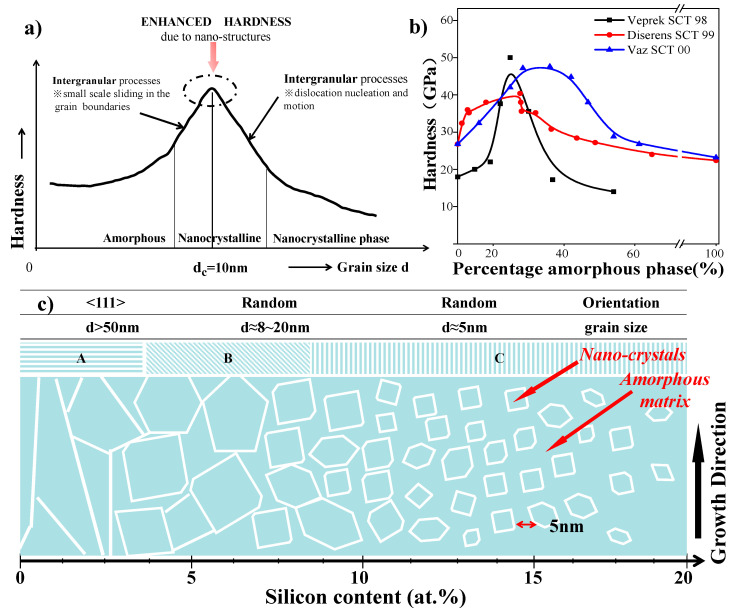
Structure-performance relations in nanocomposite coatings. (**a**) Schematic dependence of the hardness on crystallite size; (**b**) Hardness of various percentage amorphous phase; (**c**) Morphological zone model for nc-TiN/a-Si_3_N_4_ as a function of the Si content. (**a**) adapted from ref. [38]; (**b**,**c**) adapted from ref. [51].

Further research on amorphous materials has revealed their intriguing characteristics, where individual grains cannot be resolved. These materials exhibit outstanding performance attributes, such as exceptional hardness [45,56], anti-corrosion behavior [57], resistance durability [58,59], and remarkable thermal stability [50], etc. Consequently, they hold tremendous potential as ideal matrix candidates for films. The preparation of an amorphous matrix can be achieved under specific deposition parameters, and the relationship between the critical grain size (d_c_) and hardness is depicted in Figure 6. As depicted in Figure 6b, hardness is dependent on the percentage of the amorphous phase, with the highest hardness achieved at approximately 30% to 40% of the amorphous phase.

#### 2.1.2. The Composited Solid Lubricants

The pioneering work on multi-phase coatings with excellent high-temperature solid-lubricating performance was conducted by the USA National Aeronautics and Space Administration (NASA) laboratories. Over the past 40 years, they have developed the PS100, PS200, PS300, and PS400 homologous coatings, all of which demonstrate preferable solid-lubricating behavior. 

The main design concept behind PS homologous coatings is to utilize a combination of silver and fluoride as a solid lubricant, enabling the coatings to exhibit low coefficients of friction over a wide temperature range, something that cannot be achieved with a single solid lubricant [60,61,62,63,64,65,66,67]. The PS series coatings have indeed proven the effectiveness of combining lubricants. However, there is still room for improvement in terms of the hardness of the PS families. Since the current operating environment of solid-lubricating films requires continuous lubricating performance, the choice of lubricating components should also adhere to the principle of “composite”. Further details on material selection will be discussed in Section 2.2.2.

The PS304 coating, belonging to the PS coatings, has found successful applications in foil gas bearings and other high temperature sliding contacts [68]. In this context, PS304 serves as an illustrative example to demonstrate the constituent design strategy. The constituents of PS304 are carefully chosen to incorporate specific characteristics: wear resistance is derived from chromium oxide (20 wt.%), low-temperature (<450 °C) solid lubrication is provided by silver, and high-temperature (>450 °C) lubrication is achieved through the addition of 10 wt.% of BaF_2_ and CaF_2_ salts combined. These constituents are embedded within an oxidation-resistant matrix comprising Ni-(20-30) Cr powder carrier, making up 60 wt.% of the total mixture. This amalgamation of constituents further exemplifies the effectiveness of the combination principle.

#### 2.1.3. The Encapsulated Reinforcements

Indeed, combining a metal matrix with suitable reinforcements to leverage the strengths of each component is an effective approach to enhance the properties of Metal Matrix Composites (MMCs), including both strength and toughness [47,69]. This strategy draws inspiration from natural structures found in plants and other organisms [70], where different materials are ingeniously combined to achieve superior performance. 

Absolutely, the combination strategy is highly advantageous for fabricating composites and has also found successful application in the development of films [51]. Incorporating nanocrystalline and amorphous enhancers becomes an essential and logical choice when striving for multifunctional composite nitride films. This approach not only allows for excellent design flexibility, but also results in films with remarkable hardness and other advanced properties [50,51,71]. 

Furthermore, to address the issue of lubrication components, such as the soft metal element Ag, depleting during friction and leading to a sharp decline in lubrication performance, an effective solution is to encapsulate the solid lubricant within a protective enclosure, with an enhancer being the ideal choice. This can be achieved by fabricating a shell-core structure with precise deposition parameters, where the hard reinforcements serve as the protective shell, while the solid lubricants act as the core. Incorporating nano-structured and/or amorphous hard particles [3,15], as well as other hard materials like nitrides [72], carbides [73], and oxides [74], is recommended to form the protective “time-capsule” shells. The mechanisms responsible for the strengthening effects brought about by these hard inclusions will be thoroughly discussed in Section 3.3.1.

**Figure 7:**
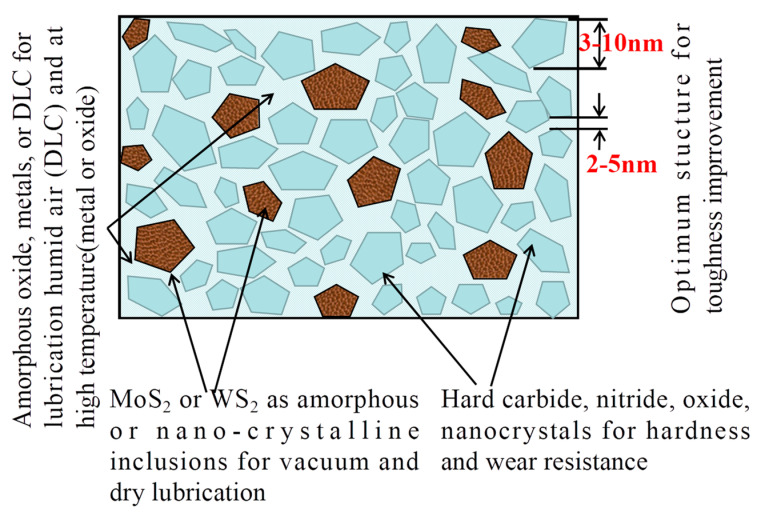
A schematic diagram of a conceptual design of functional coating with nanocrystalline phases embedding in an amorphous matrix, adapted from ref. [3].

S. Veprek introduced the concept of superhard coatings with nanocrystalline inclusions and an amorphous matrix [45,75,76,77,78,79,80,81,82,83,84]. According to his belief, the grains in the nano-composite coating contain either no crystal defects or only a small amount, resulting in hardness and volume elastic modulus values that are close to the theoretical limits. This is in stark contrast to typical materials, which exhibit volume elastic modulus values only about 1/100 of the theoretical limit. Building upon this strategy, S. Veprek and coworkers successfully prepared a nano-composite coating (nc-TiN/α-Si_3_N_4_/α-&nc-TiSi_2_) with a hardness over 105 GPa in 2000, boasting a remarkable hardness exceeding 105 GPa, thus posing a significant challenge to diamond’s status as the most rigid material. Figure 7 visually represents the design strategy: (1) the amorphous phase serves as the matrix, providing structural integrity to the film (the writer himself referred to this component as the lubricant at high temperatures); (2) the amorphous or nano-crystals function as lubricating inclusions embedded within the matrix; (3) and the nano-crystals act as reinforcements.

Based on the discussions above, it is evident that the “amorphous matrix + inclusions” design strategy is indeed a viable approach to fabricate nitride films with exceptional hardness and outstanding lubricating performance. Considering the existing combinations found in nitride composite films, the phase constitutions involving nano/non-crystalline inclusions embedded in the amorphous matrix also yield positive results [3,51,52,85,86]. Furthermore, it is certain that other design strategies can also be tailored to achieve specific properties of films. For instance, the composition gradient-transition layer [87] and the stabilized entropy strategy achieved through alloying the films [45,88,89,90] are viable methods to customize film properties. In addition, various engineering strategies are available to facilitate the fabrication process. These include the confined synthesis method [91], the template-assisted method [86], and the self-assembly method, among others.

### 2.2. The Chemical Constitutions

**Figure 8:**
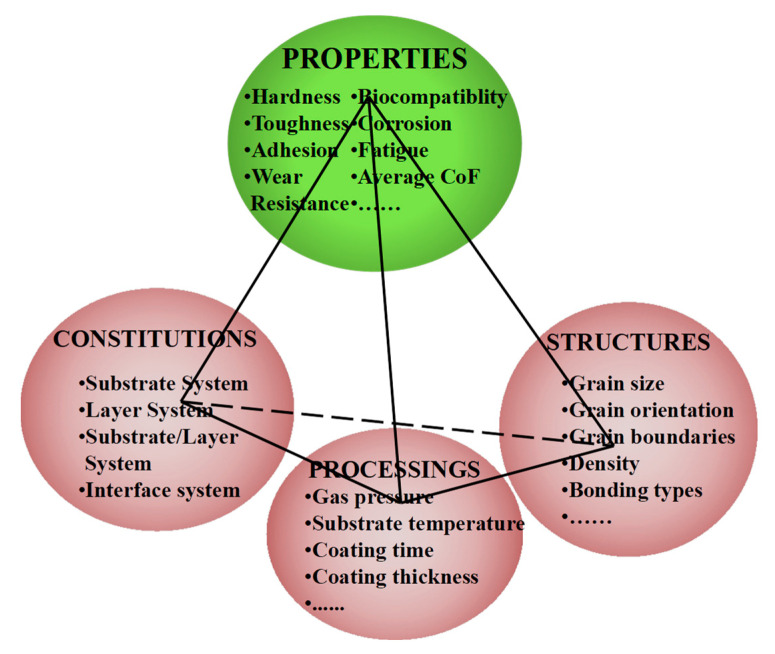
The processing-properties-performance-constitution relation between various fundamental parameters of a coating system.

The thermal stability of films is not only dependent on the films themselves but is also influenced by the thermal stability of the substrate. As discussed extensively in Section 2.1, the “amorphous effect + combination” design strategy has been thoroughly explored. In this section, our focus will be on summarizing the target materials that have an impact on the properties and service performance of the films. Subramanian et al. [92,93,94] have provided a comprehensive summary of multi-component and multilayer coatings, revealing the inter-relationship between various fundamental parameters within a coating system, as depicted in Figure 8.

#### 2.2.1. The Matrix Film

The chemical composition of nitride films is primarily determined by the deposition process, the choice of target materials [95], the nitrogen flux [96], the negative bias [97], the vacuum degree, and other relevant factors. In this section, we will specifically discuss the target materials used for the matrix, the lubricants, and the reinforcements in nitride films. Typically, nitride films are synthesized through the reaction of transition metals with nitrogen (N_2_) gas, along with the inclusion of other necessary components.

**Figure 9:**
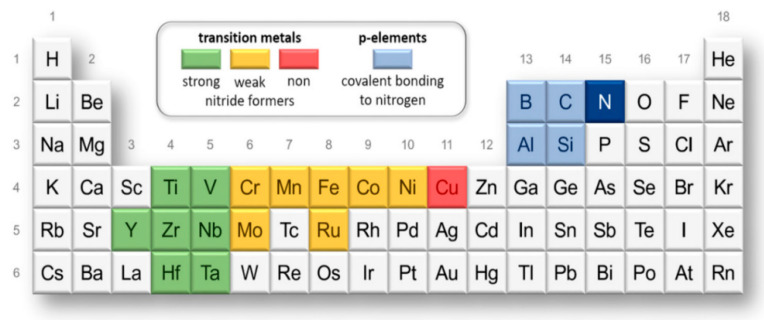
The processing-properties-performance-constitution relation between various fundamental parameters of a coating system, adapted from ref. [98].

As depicted in Figure 9, the transition metals can be categorized into different groups based on their nitride-forming capabilities. These groups include strong nitride formers, such as Y, Ti, Zr, Hf, V, Nb, and Ta; weak nitride formers, including Cr, Mo, Mn, Fe, Ru, Co, and Ni; P-elements, which form covalent bonds with nitrogen, like B, C, Al, and Si; and non-nitride former, represented by Cu [98]. When selecting the matrix materials for nitride films, it is crucial to consider the specific properties and the intended service performance of the films. The choice of matrix materials should align with the desired functional characteristics and overall performance requirements of the films. 

**Figure 10:**
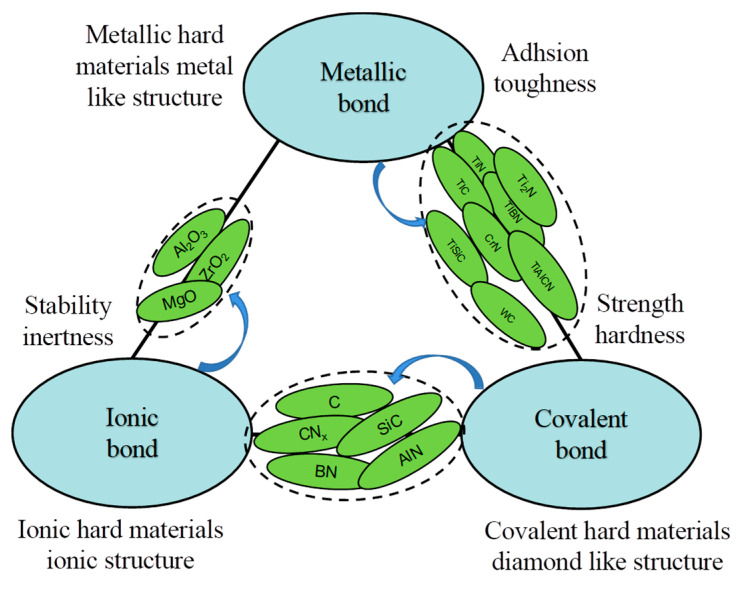
Hard materials for nanocomposite coatings in the bond triangle, adapted from ref. [99].

In Figure 10, three types of chemical bonds are evident [99]: metallic, covalent, and ionic. Each bond type possesses distinct advantages, differing from the others. Metallic bonds are advantageous for adhesion and toughness, covalent bonds offer hardness and strength, and ionic bonds provide stability and inertness.

Indeed, the formations of ionic bonds, covalent bonds, and metallic bonds involving the transition metals and N_2_ occur under different synthesis conditions. Notably, compounds with covalent chemical bonds, such as α-Si_3_N_4_, α-BN, and α-CNx, typically exhibit premium hardness. These covalent compounds are crucial as the matrix component for the films and offer great potentials and advantages over other bonding types. The unique properties of covalently bonded compounds make them highly desirable for serving as the matrix material in films. Their exceptional hardness provides the necessary foundation for achieving films with superior mechanical properties and performance characteristics [100,101,102].

#### 2.2.2. The Solid Lubricants

Absolutely, selecting the appropriate target materials for lubricants is of utmost importance, especially when considering the specific operating conditions. The choice of lubricants should align with the requirements of the application, such as temperature, pressure, speed, and environmental factors. Properly chosen lubricants can significantly impact the overall performance, efficiency, and durability of the system or equipment. Indeed, research studies [103,104] have highlighted that relying on a single lubricating phase to cover a wide temperature range, from room temperature (RT) to elevated temperatures, is not a feasible approach. Single-phase lubricants tend to perform optimally only within their specific temperature zones. In Section 4, we will provide detailed explanations and interpretations of the lubricating mechanisms involved.

**Figure 11:**
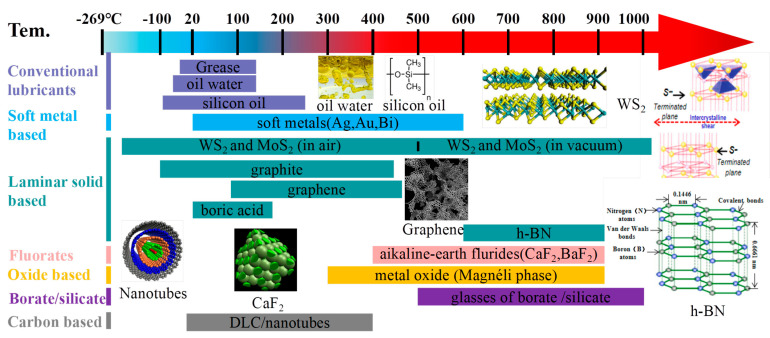
A graphical representation of effective temperature ranges for solid-lubricating materials, adapted from ref. [105].

Figure 11 visually represents a graphical depiction of several solid lubricating materials, along with their recommended working temperature ranges. This illustration clearly demonstrates the diverse lubrication stages and highlights the limitations of single-phase lubricants in operating over a broad temperature spectrum. By understanding the specific lubrication mechanisms at different temperature ranges, researchers can gain insights into developing tailored lubrication strategies that are more effective and suitable for a wide range of operating conditions [105]. In the temperature range from RT to 500 °C, it is recommended to choose materials with 2D-lamellared structures, such as graphite, graphene, and WS_2_, for lubrication purposes. Additionally, Diamond-like Carbon (DLC) films are known to exhibit exceptional lubricating properties due to their hardness, making them a favorable option. Moreover, soft metals such as c-Cu, c-Ag, and c-Ni are also suggested for consideration [106,107,108].

It is widely recognized that when the ambient temperature is below 500 °C, the lubricating performance is primarily influenced by the nature of lubricants. As the operating temperature increases, the lubricant present in the film will decrease due to ablation and diffusion, ultimately leading to wear of the films as the lubricant becomes depleted. In the service environment, the temperature is predicted to reach 800 °C or even higher, owing to the heat generated from friction between the contact surfaces [109,110,111]. Hence, it becomes imperative to establish a continuous lubrication requirement when designing the film compositions of lubricants. The incorporation of solid lubricating films for temperatures ranging from 300 °C to 1000 °C will be discussed next. In the temperature range of 300 °C to 850 °C, it is recommended to consider Magnéli phase-forming elements, alkali metal fluorides, and certain low-melting-point metals. For the temperature range of 600 °C to 1000 °C, inorganic oxygenates and/or fluorates, as well as high melting point metal oxides with lubricating properties, are advised. Detailed elucidation of the mechanisms involved will be provided in Section 4. 

#### 2.2.3. The Reinforcements

Exactly, in nano-composite films, all film components that possess sufficient hardness can serve as reinforcements [3,15,72,73,74], just as illustrated by the nano-particles in Figure 7. These components include nano-structured carbides, oxides, nitrides of metals, cermets, and cubic boron nitride, such as SiN_x_, BN_x_, AlN [112,113], CN_x_ [114], TiSi_x_, TiB_2_, WC, α-Al_2_O_3_, ZrO, and so forth. The incorporation of such hard nano-structured materials as reinforcements enables nano-composite films to achieve enhanced mechanical properties and improved performance, making them highly valuable in various applications. On the other hand, enhancers [115,116,117,118] are not limited to individual particles; they can also act as enhancers when carbides, nitrides, or oxides are deposited alternately, layer by layer, at nanoscale thickness, forming gradients within the film. Films with gradient phase components demonstrate desirable properties, including enhanced hardness, improved thermal stability, and increased oxidation resistance. The incorporation of gradient phase components in nano-composite films allows for the fine-tuning of their properties, enabling the films to meet specific performance requirements for various applications. 

## 3. The Strengthening Mechanisms

As solid lubricating films, traditional nitride materials (TiN [10], CrN [11], ZrN [12], etc.) are unable to meet all the requirements. Over time, the comprehensive performance of nitride films has been steadily improving, progressing from 1st Generation to 4th Generation films, driven by various strengthening mechanisms, such as doped additions and architectured microstructures. These strengthening mechanisms are not limited to superhard films but are applicable to all films, including both hard and superhard films.

It is important to note that the phenomenon of reinforcement is extremely complex, and there is no single rule that can fully explain its internal principles. In this article, we will delve into the strengthening mechanisms in detail, covering Section 3.1, Section 3.2, Section 3.3, Section 3.4, Section 3.5, Section 3.6 and Section 3.7, to provide a comprehensive understanding of the topic. 

### 3.1. The Solid Solution Mechanisms

**Figure 12:**
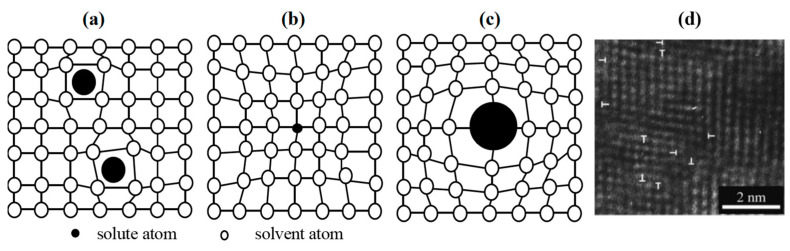
Schematic of dislocation triggered by solid solution; (**a**) interstitial solid solution; (**b**,**c**) substitution solid solution; (**d**) HRTEM micrograph showing misfit dislocations, adapted from ref. [119].

The solute atoms dissolve into the interstitial sites of the crystals, causing distortions [119] within the crystal lattice, resulting in the formation of either a one-phase super-saturated crystalline matrix or a nano/noncrystalline matrix with a certain solubility of the doped elements. This phenomenon induces solid solution strengthening [50,60,120], as illustrated in Figure 12. The doped atoms can replace the original ones in the lattice, leading to the gradual accumulation of regional distortions due to differences in atom sizes. These lattice distortions impede dislocation motions, thus enhancing the strength and hardness, making solid solution strengthening the primary reason for the observed strengthening effect. 

The solid solution hardening effect, arising from stress contribution and the hindrance of dislocation motions, can be expressed as follows (Equation (2)) [121].

(2) Δτ=Gb→(c)12(ε)32

where G is the shear modulus (GPa), $\vec{b}$ is Burger’s vector (nm), c is the concentration of solute atoms, and ε is the lattice microstrain owning to solute atoms.

The solid solution phenomena will be introduced in the following two cases, during the deposition [122] and the deformation [123].

#### 3.1.1. The Solid Solution Mechanisms Based on Solubility

The solid solution strengthening discussed here occurs as a metastable stage during the unbalanced growth of the deposition process. At times, the atoms may not possess enough energy to effectively spread and diffuse on the substrate during deposition. As a result, a large number of defects, such as vacancies, can be generated in the crystal lattices. Simultaneously, the doped atoms dissolve into the matrix films, leading to the formation of either interstitial solid solution or substitutional solid solution. In this paper [124], the formation process of the amorphous Si_3_N_4_ phase is elucidated, and a structural evolution model of the VSiN ternary nitride film is established. The model exhibits three distinct stages, with the boundary changing from fuzzy to clear as the solubility of doped Si atoms undergoes variation.

Junhua Xu and his co-workers [122] developed a three-step model and proposed a four-step model to explain the structure evolution of binary VN films. Due to the low deposition temperature at RT and the N_2_ flow rate, the atoms lack sufficient energy to spread effectively on the substrate during the deposition process. As a result, a large number of defects, including vacancies, dislocations, and sub-boundaries, exist in the crystal lattice, constituting the first step in the structure evolution model. Håkan W.H. et al. [125] argue that hardness is the resistance against localized plastic deformation, which corresponds to the motion of dislocations at a microscopic level. Indeed, a crystal lattice with numerous defects will hinder the motion of dislocations, thereby enhancing the hardness of films in comparison to films with fewer defects or dislocations.

#### 3.1.2. The Solid Solution Mechanisms Based on Deformation

**Figure 13:**
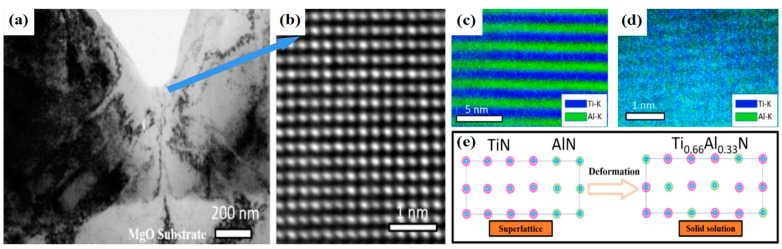
Schematic atomic model illustration of the intermixing process, where the rs-TiN/rs-AlN SL evolves to a Ti_0.67_Al_0.33_N solid solution. (**a**) A HRTEM image of the deformation zone; (**b**) A cross-sectional HAADF image; The elemental mapping (EDXS) at the position (**c**) far away from the impression surface and (**d**) in the surface region of the impression. (**e**) A schematic atomic model illustration of the intermixing process. Adapted from ref. [123].

In this section, we will provide an explanation of the process of deformation-induced solid solution. Recent studies have demonstrated that deformation can trigger phase transformation in AlN or Al alloyed TMN [126,127,128,129,130], as supported by simulations using AIMD, DFT, and classical MD methods, as shown in Figure 13. This indicates that under mechanical load, the solute atoms can be pushed into the crystalline lattice, resulting in the formation of a nano-scaled solid solution. It is important to note that the deformation-induced intermixed solid solution is more likely to form in the surface regions rather than the inner regions of the material. Zhuo Chen et al. [123] reported their findings of an “inter-mixed” region when conducting indentation on a single-crystalline superlattice. In this region, the layer structure underwent a transformation into a solid solution under applied loads, as opposed to the polycrystalline superlattice. The researchers declared that this interfacial mixing was initiated by the nanoindentation process. Zhuo Chen et al. [131] revealed several phase transformations in AlN, which encompassed processes such as B4 to B1, B3 to B1, B1 to B3, and B1 to B4, occurring under various deformation processes such as indentation, tension, and shear. Furthermore, previous observations of local phase transformation experiment (TEM results of indented TiN/AlN superlattice [127] and ZrN/Zr_0.63_Al_0.37_N superlattice [132]) further confirmed that the deformation indeed triggers phase transformations. 

### 3.2. The Grain Size Effect

#### 3.2.1. The Grain Refinement Mechanisms

As discussed in detail in Section 2.1.1, the non/nanocrystal phases are highly promising candidates that offer significant advantages over traditional materials. One of the main distinguishing features of these nanocomposites is their enhanced hardness. Subsequent studies have demonstrated that the properties of nanostructured composite films can be further improved through cooperative deformation, where grain sizes typically range from 3 to 10 nm, roughly matching or slightly exceeding those found on grain boundaries. Grain boundaries play a crucial role in facilitating the formation of dislocations and promoting sliding during plastic deformation [3]. This is mainly due to the following reasons:

(1) The grains in the films are fine, making deformation more challenging within the grains.
(2) The presence of numerous grain boundaries with 3D skeletal structures impedes the motion of dislocations, resulting in improved hardness and toughness. Additionally, the films can release deformations through the sliding of grain boundaries.

Based on these mechanisms, the Veprek Model of hard films was later proposed, providing valuable guidance for the design of films with exceptional hardness.

Uniform fine grains in the film help release stress concentration, enabling the film to better accommodate deformation and, as a result, enhancing both hardness and toughness. Furthermore, J. Musil et al. [38] revealed that when the grain size is smaller than approximately 10 nm, or about 3–5 monolayers, the atomic force becomes involved in the formation of sub-boundaries. This leads to a range of grain size-related strengthening effects, including: (1) dislocation-induced plastic deformation, (2) the unique nanostructure of materials, and (3) cohesive forces between atoms. These factors collectively contribute to the exceptional properties exhibited by nanocrystal films, such as super hardness, toughness, thermal stability, inoxidizability, and anti-wear behavior.

Hugosson et al. [125] demonstrated that a superlattice structured film, consisting of layers with a nano-size period λ and a strong interlayer within 3~5 monolayers (ML), could further enhance the hardness of the film. Additionally, they found that the grain size effect (or Hall-Petch relation) [133,134] (Equation (3)) is applicable to both the monolayered and multilayered films.

(3) $$H=H_{0}+{\mathrm{Kd}}^{n}$$

where H is the hardness (GPa), H_0_ is the original hardness (GPa), K is a material constant, d is the grain size (nm), *n* is the grain size exponent (typically −1/2).

What is more, a previous study [135] reveals that when the grain size of thin films is less than 20 nm, the number of dislocations in one grain can be estimated using the following equation (Equation (4)). When an appropriate number of dislocations (*N*) is present, grain rotation and grain boundary sliding can become the primary deformation mechanisms.

(4) $$N=\frac{k\pi \tau D}{4G\vec{b}}$$

where *N* is the number of dislocation in one grain, k is a constant close to unity, *τ* is the critical resolved shear stress (GPa), *D* is the grain size (nm), *G* is the shear modulus (nm), and $\vec{b}$ is the magnitude of Burger’s vector (nm).

#### 3.2.2. The Internal Interfaces

**Figure 14:**
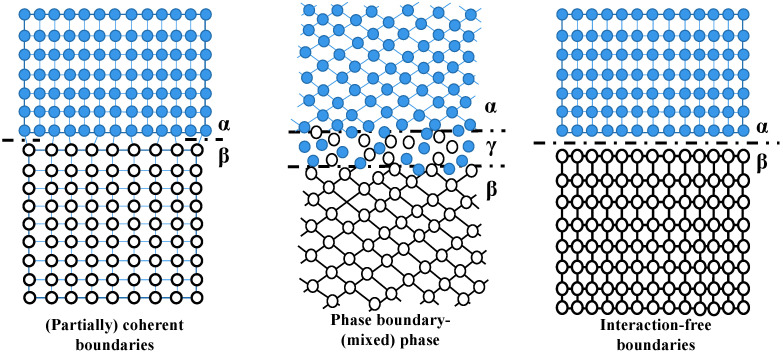
Different natures of interfaces in multi-coatings or at the substrate/layer interfacial, α, β, γ represents different crystal structures, adapted from ref. [92].

Boundaries in the context of this study encompass phase boundaries, grain boundaries, layer interfaces referred to as “interfaces” in the text, and boundaries between chemical separation domains. All of these are collectively known as “internal interfaces”, as shown in Figure 14. As previously mentioned, microstructures such as crystal boundaries play a dominant role in inhibiting crack extension, blocking the movement of dislocations, and coordinating deformations of films. This is one of the main reasons for the enhancement of hardness. Additionally, non-coherent phase boundaries, similar to large-angle crystal boundaries, are also generated during the deposition process. It is believed that the better plasticity observed in the polycrystalline sliding lattice can be attributed to its large-scale grain boundary sliding deformation behavior [131].

Carsley et al. [136] developed a straightforward phenomenological model utilizing empirical fitting parameters for the “hardness” of grain boundaries and their thickness. This model enables the fitting of experimental data regarding the dependence of hardness on crystallite size, volume fraction, and grain boundaries, according to the following formula (Equation (5)) [136,137,138]:

(5) $$H(d)=f_{c}(H_{0}+\beta \cdot d^{-0.5})+(1-f_{c})\cdot H_{G\cdot B}$$

where f_C_ and (1 − f_C_) are the volume fraction of the crystalline material and the grain boundaries, respectively; H_0_ (GPa) is the hardness of coarse-grained material and H_G.B_ (GPa) is the ”hardness” of the grain boundaries while β is a material constant.

Cruz. R. et al. [139] revealed that, based on Koehler hardening, dislocations are repelled by the interfaces in the films between components, which is further influenced by the contribution of the elastic modulus difference between the components. Similarly, G. Abadias et al. [140] reported that the increase in hardness can be attributed to a hardening effect resulting from the presence of a large number of interfaces parallel to the substrate surface in multilayered coatings. Earlier literature [139,140] has confirmed the significant contribution of internal interfaces to the enhancement of hardness. In conclusion, we believe that the internal interface theory, encompassing coharmonic and non-coharmonic boundary theories, can effectively explain the strengthening phenomenon observed in films, regardless of the type of boundary formed during deposition.

#### 3.2.3. The Isotropic Effect

The isotropic effect, also known as the Inverse Hall-Petch Relationship, is a typical characteristic observed in amorphous materials [60,141]. To provide more clarity, we will elaborate on the isotropic effect based on the model where nanocrystals are encapsulated in amorphous matrix films, similar to the nc-TiN/α-Si_3_N_4_ model proposed by S. Veprek (or Model I, as proposed in Section 2). The hardness enhancement in this model can be attributed to the following reasons.

The isotropic effect of the amorphous phase plays a crucial role in strengthening the nc-/α-structured films. It aids in restricting the propagation and deflection of cracks by facilitating energy dissipation in the amorphous nitride films. The hardness of amorphous films surpasses that of normal-size nanocrystal films due to the size effect [38,59], as illustrated in Figure 6a. The “strongest size” *d_c_*, which is approximately 10–15 nm, is applicable only for well-consolidated materials with low porosity, such as films deposited by CVD or PVD. For nanocrystalline materials prepared by consolidation, the maximum hardness may appear at a higher crystallite size.

Amorphous films can undergo a phase transformation stage under appropriate conditions, such as ambient temperatures. This transformation can induce both nucleation and growth stages, leading to the storage of energy in the form of residual stress and the formation of a large number of structural defects. Additionally, the coordinating deformation and precipitation hardening mechanisms are also suitable for amorphous films [131,142]. Therefore, the hardness enhancement due to the isotropic effect is a complex process involving various factors, including energy relaxation, subgrain boundary (and/or sub-boundary sliding), phase transformation, coordinating deformation, and precipitations [142].

### 3.3. The Secondary Phase Strengthening Mechanisms

**Figure 15:**
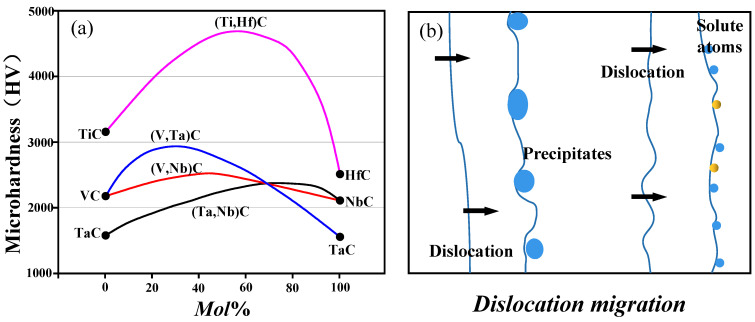
(**a**) Microhardness of mixed carbides (precipitation hardening) (Adapted from ref. [92]); (**b**) Schematic illustration on how dislocations are held up at precipitates and solutes.

Secondary phase strengthening [60,143,144,145], also known as precipitation strengthening, plays a dominant role when tiny and dispersed nanoparticles precipitate from the primary matrix phase. As shown in Figure 15, a strengthening effect can occur through phase separation by cooling down a ternary mixed phase with a suitable miscibility gap. For instance, taking (Ti,Hf)C as an example, mixed crystal hardening (or precipitation hardening) is demonstrated in Figure 15. When the mol% of HfC accounts for 50% in the mixed carbide (Ti,Hf)C, the microhardness reaches its peak, and it increases by approximately 50% more than that of pure TiC. Accordingly, the possible strengthening (dispersion strengthening) contributing to H enhancement would be semi-quantitatively evaluated (and $H≅1/3\sigma_{ys}$) [146], primarily attributed to the Orowan bypass mechanism [47,86]. The increase in yield strength resulting from the Orowan bypass mechanism can be estimated by the following expression [147].

(6) $${\Delta \sigma }_{or}=0.4M\left(\frac{Gb}{\pi \lambda }\right)\left(\ln\frac{2r}{b}\right){\left(1-\nu \right)}^{-0.5}$$

where ν is Poisson’s ratio, λ is the inter-precipitate distance (nm), and r (nm) is the mean radius of a circular cross-section in a random plane for a spherical precipitate.

#### 3.3.1. The Precipitations Forming during Deposition

Nano-composite coatings composed of small amounts of nanograins dispersed in an amorphous matrix are referred to as DNG/AM nano-composites. These nano-composites can be formed during the deposition process, with nanograins dispersed within the matrix [38]. Additionally, the solubility of the doped atoms dissolving into the matrix lattice is also a crucial factor. When the solubility of the dopants reaches saturation, the additive atoms will form chemical compounds with reactive gas, and these chemical compounds will aggregate at the grain boundaries, resulting in the formation of structures with precipitating particles embedded in the matrix [3,51,52,85,148].

**Figure 16:**
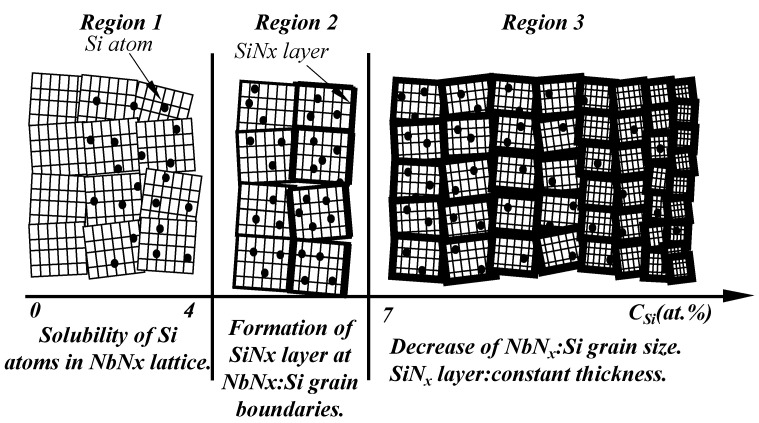
Schematic illustration of the secondary phase forming during deposition, adapted from ref. [124].

The particles embedded in the matrix act as reinforcements [72,73,74], and they intercept the dislocation motions through interactions with the dislocations [121]. These reinforcements and interactions contribute to enhancing the mechanical properties, including hardness, which highlights the advantages of combinations and multi-phases [94]. In Figure 16, a vivid schematic illustration is presented, showing that with the increase of Si atoms embedded in the NbNx matrix, a SiNx layer forms at the SiNx:Si grain boundaries. Interestingly, the unit cell (NbNx:Si grain size) decreases while the thickness of the SiNx layer remains constant.

#### 3.3.2. The Precipitations Forming during Post-Deposition

**Figure 17:**
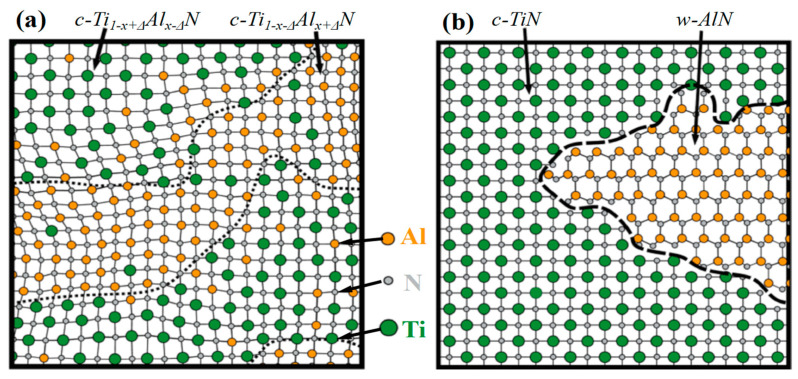
Schematic illustration of the atomic assembly for (**a**) the isostructural decomposition into c-TiN- and c-AlN-enriched domains which causes the strain increase in the single-phase regime, and (**b**) the dual phase structure at higher T_a_ resulting in strain decrease due to grain growth, adapted from ref. [150].

The spinodal decomposition [86], which occurs during the annealing treatment, is an important strengthening phenomenon. During this decomposition process, the doped atoms possess enough energy, and the high annealing temperature facilitates easier diffusion of the doped atoms compared to the deposition treatment period. The strengthening mechanisms are triggered by the spinodal decomposition of metastable phases, where the precipitations aggregate at the grain boundaries. Chen W.L. et al. [149] proposed a two-step decomposition mechanism for the c-AlMeN solid solution, which is commonly accepted. In the first step, the coherently enriched-Me and enriched-Al fcc-domains form through spinodal decomposition, leading to aging hardening. Then, in the second step, the stable formation of HCP-AlN occurs through nucleation and growth mechanisms, with a slight loss in hardness. The HCP-AlN gradually aggregates at the grain boundaries, forming 3D-architectured microstructures and/or self-assembled nano-precipitations embedded in the matrix. Once the spinodal decompositions seemingly reach an end, dislocation motions or plastic deformation are hindered, leading to an enhancement in hardness. The spinodal decomposition process is illustrated in Figure 17.

### 3.4. The Strengthening Mechanisms Induced by Stress Field

#### 3.4.1. Residual Stress

Residual stress engineering can be utilized to enhance film performance through the application of strain hardening. Additionally, it aids in improving erosion resistance, with fracture dominating the erosion process, by carefully inducing a controlled amount of compressive stress in hard and brittle ceramic coatings. As indicated in previous studies, residual stress is generated during film growth [151]. The residual stress ($\sigma_{Re}$) of the films prepared by PVD consists of two main components: residual thermal stress ($\sigma_{T}$) and residual internal stress ($\sigma_{I}$). The residual stress [152] can be calculated using the following equation (Equation (7)):

(7) $$\sigma_{Re}=\sigma_{T}+\sigma_{I}$$

The residual stress $\sigma_{Re}$ (Gpa) can be calculated by Stoney’s formula (Equation (8)) [6,153]:

(8) σRe=Ests2(1r−1R)/6(1−νs)tf

where the subscript s refers to the substrate; $E_{s}$ (Gpa) and $\nu_{s}$ are Young’s modulus and Poisson’s ratio of substrate, respectively; t_f_ and t_f_ are the thickness (mm) of film and substrate, respectively; r and R are the curvature radii of coating and primary substrate, respectively. The thermal stress (Gpa) ($\sigma_{T}$) can be enumerated as Formula (9) [120,154]:

(9) $$\sigma_{T}=\frac{E_{f}}{1-\nu_{s}}(\alpha_{f}-\alpha_{s})(T_{D}-T_{M})$$

where E_f_ is the elastic modulus (Gpa) of the film; $\nu_{s}$ is the Poisson’s ratio; α_f_ is the thermal expansion coefficient (TEC) of the film; α_s_ is the TEC of the substrate; T_D_ is the temperature of film deposition; T_M_ (°C) is the temperature of testing the residual tress.

Generally, films with high hardness values are accompanied by high residual stress levels. Previous findings [155,156,157] have shown that compressive stress (negative) tends to improve both the hardness and tribological behavior of coatings, while tensile stress (positive) reduces them. However, it is important to note that high (tensile) residual stress is the primary cause of film delamination, substrate plastic deformation, and cracking during the deposition process. Therefore, it is crucial to control the residual stress in films [155]. Meanwhile, compressive residual stress plays a crucial role in enhancing hardness. Introducing mechanical compressive stresses into coatings leads to hardness enhancement, which is a contributing factor to the increased hardness. R. David et al. [156] studied the depth distribution of residual stress in TiN coatings prepared by ion implantation and proposed a Gaussian distribution to describe the depth distribution of residual stress in grazing incident X-ray diffraction. Coatings with higher hardness than that of bulk materials can be synthesized based on the high ionic energy, resulting in a large number of defects and consequent strain hardening. However, strain hardening is often considered to have a minor contribution to the extremely high hardness values of nano-composites due to the easy migration of defects to phase boundaries during growth.

Most coatings prepared by PVD are prone to developing residual (compressive) stress, and these residual stresses have a significant impact on coating hardness. Karlsson et al. suggested [151] that the residual stresses in TiN and TiC_x_N_1−x_ films mainly result from structural defects such as solute atoms, point defects, line defects, and area defects. These defects hinder dislocation movements, leading to an increase in film hardness. This mechanism is considered the primary reason for the hardness enhancement of films. This conclusion finds support in the near-linear relationship between hardness and residual (internal) stress observed in arc-deposited TiC_x_N_1−x_ films [157]. A defect hardening mechanism was proposed [157], illustrated through a schematic representation of the contributions to hardness, which includes the defect hardening mechanism, grain boundary hardening mechanism, and intrinsic hardness, respectively. Moreover, Karlsson and his co-workers believed that the residual (internal) stress was proportional to strain [158]. C.A. Davis et al. [159] proposed a simple model to predict the residual stresses in arc-evaporated TiN films. The model incorporates the competition between two different rates: the defect creation rate and the defect annihilation rate under steady-state conditions.

**Figure 18:**
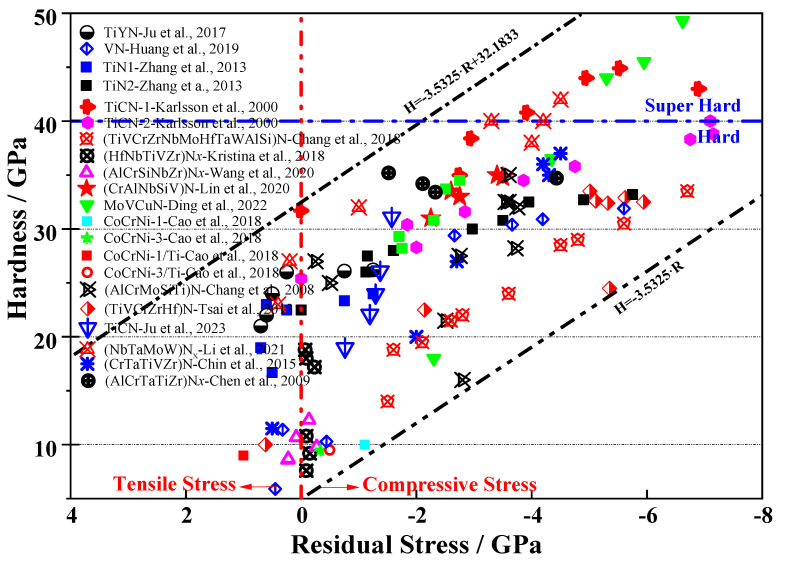
Residual stress vs. hardness of various nitride coatings (Data from refs. [7,135,153,157,161,162,163,164,165,166,167,168,169,170,171,172].

When compressive stress dominates the residual internal stress, there is a dramatic improvement in the hardness value of the coatings. Junhua Xu also reached a similar conclusion regarding the Ti-Y-O-N film, where the hardness showed a positive correlation with residual stress [7]. Additionally, several other coatings have shown an apparent linear relationship between residual stress and hardness. Compressive residual stress enhances the hardness, while tensile stress impairs hardness (as depicted in Figure 18). For example, coatings such as TiN, Ti(C,N) [157], or CrN [160] have exhibited this behavior. On the contrary, when tensile stress becomes dominant over compressive stress in films, issues arise such as film delamination from the substrate and crack propagation where the tensile stress concentrates. These conditions lead to failures of the films and indicate that tensile residual stress is harmful to the coatings.

#### 3.4.2. The Alternating Stress of Superlattice Structured Films

In 1970, Koehler introduced the concept of designing what is now known as a heterostructure or superlattice film. In this structure, alternating layers of two or more materials, denoted as A and B for example, are deposited on a substrate with a specific period. Generally speaking, the chosen materials [173,174,175,176,177] must meet certain criteria. Firstly, there should be strong bonding between A and B atoms, comparable to the bonding between two A atoms or two B atoms. Secondly, while having the same crystal structures is not mandatory, it becomes more intriguing when A and B have different crystal structures. Additionally, it is desirable for the thermal expansion coefficient (TEC) to be as similar as possible to maintain sharp interfaces, preventing changes in temperatures from disrupting the coherent relationships of A and B. Thirdly, at the deposition temperature, the lattice parameters should be nearly equal. Fourthly, it is beneficial for the elastic constants (or shear modulus) to differ significantly, as this leads to varying required line energies of dislocations in large single crystals of materials. Lastly, the modulation period should be in the nanometer range. The superlattice nitride films present an environment where dislocation motions can be hindered due to the nano-scale thickness of 2~3 nm. Koehler et al. [175] suggested that nickel and copper are one possibility, and the films with superlattice structures possess a pronounced hardness of about 40 GPa. Hardness (H) or strength (σ) (The Vickers hardness is further approximated by H ≈ 3σ [178], where σ is the yield stress.) increment caused by shear modulus’ differences ([86,123,142,147] can be given by Equation (10)):

(10) $${\Delta \sigma }_{mod}=0.0055M{\left(\Delta G\right)}^{3/2}{\left(\frac{2\Phi }{Gb^{2}}\right)}^{1/2}{b\left(\frac{\langle R\rangle }{b}\right)}^{\left(\frac{3m}{2}-1\right)}$$

where M is the mean orientation factor; b is the magnitude of Burgers vector (nm); Φ is the volume fraction estimated by considering the additives as ideal spheres; $\langle R\rangle$ is the mean radius (nm); G is the shear modulus (GPa); ΔG is the shear modulus mismatch (GPa); and m is a constant.

Junhua Xu and colleagues [179] conducted a study on the microstructure and superhardness effect of superlattice films. They proposed that the main reason for the enhanced hardness of these films is the presence of alternating stress fields resulting from interfacial coherent strain. Furthermore, it is worth noting that previous research [86,180,181,182,183] extensively supports the notion that superlattice structures can simultaneously improve both the hardness and fracture toughness of ceramic films. This indicates the significant potential of superlattice films in enhancing mechanical properties, making them highly promising for various applications.

### 3.5. The Template Effect

In modern times, it has become commonplace to deposit multi-component films or multilayered films, mainly because of their advantageous mechanical properties and lubricating behaviors. However, when two crystals with different structures come together, structure transition regions can occur. These transition regions can lead to abnormal phenomena due to the “template effect” [86,184,185,186,187,188], also known as the Epitaxial Stabilization Effect. The template effect is responsible for causing two materials that should have different structures to form the same tissue structure during the deposition process. 

**Figure 19:**
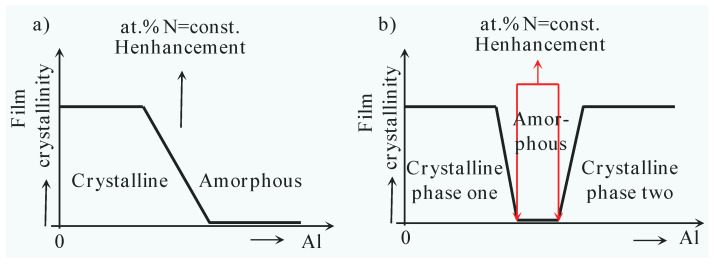
Schematic illustration of three transition regions of A_1−x_B_x_N compounds: (**a**) Transition from crystalline to amorphous phase, (**b**) Transition between crystalline phases of two different materials, e.g., Ti_1−x_Al_x_N results in TiN nitride for x = 0 and AlN nitride for x = 1. Adapted from ref. [38].

The transition regions of A_1−x_B_x_N compounds were shown in Figure 19 (Adapted from ref. [38]), from which we can see a distinct H enhancement between different structures due to the coherent growth (template effect). Zhang Kan and coworkers [86] proposed that a core@shell-like nanocomposite film can be deposited using the template effect. They revealed that thin Ta-shells are likely to form a pseudo-crystal structure under the template effect of the inner TaC nanocrystalline, maintaining the same structure as TaC-cores. The phenomenon of coherent growth is predominantly influenced by the minimization of interface energy and the layer thickness. Overall, the template effect plays a crucial role in creating unique structures in multi-component films or multilayered films, leading to enhanced mechanical properties and interesting material behaviors. What is more, the coherency strengthening [86,142,147,189,190,191] between the TaC-cores and Ta-shells can be described as Equation (11):

(11) $${\Delta \sigma }_{coh}=M\alpha {\left(G\varepsilon \right)}^{3/2}{\left(\frac{\langle R\rangle \Phi }{0.5Gb}\right)}^{1/2}$$

where α is a constant; ε is the mismatch parameter; $\langle R\rangle$ is the mean radius; Φ is the volume fraction estimated by considering the additives as ideal spheres; G is the shear modulus, b is the magnitude of Burgers vector.

### 3.6. The Valence Electron Concentration Effect

In recent times, there has been significant progress in improving the mechanical properties of films by adjusting the electron concentrations. The concept of sub-stoichiometric compounds for certain transition metals was proposed by Magnéli in 1953. This concept aligns with recent reports that highlight the importance of valence electron concentration (VEC) [24,92,125,144,145,192,193] as a crucial physical parameter influencing the phase composition of chemical compounds. J. Alan et al. [142] reported that the motion of dislocations in films is hindered by precipitate particles through various interaction mechanisms. One such mechanism is chemical strengthening, where the hardness of films is enhanced as a result. This means that by manipulating the electron concentrations and phase composition of chemical compounds, it is possible to improve the hardness and mechanical properties of films, making them more suitable for a wide range of applications. 

**Figure 20:**
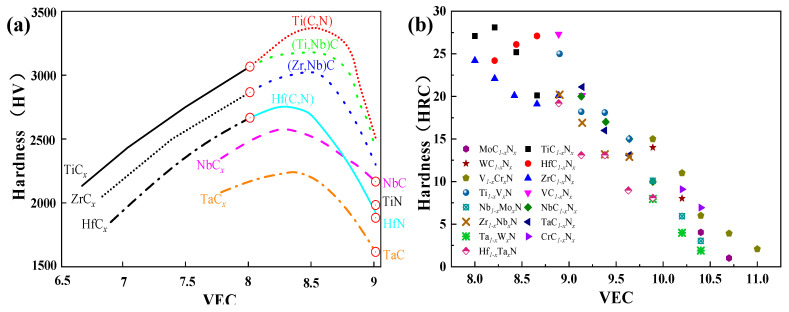
Microhardness of carbides, mixed carbides, or carbonitrides as a function of the VEC, (**a**) adapted from ref. [92], (**b**) adapted from ref. [194].

Figure 20 illustrates the relationship between valence electron concentration (VEC) and the hardness of carbides, mixed carbides, or carbonitrides. Interestingly, at a specific VEC value of approximately 8.4, the materials exhibit maximum hardness. This optimal VEC value can be achieved in binary compounds by adjusting the stoichiometry [92,151,193], and in ternary mixed crystals through substitutions in either the metal or nonmetal sublattice. The VEC effect [194] is clearly demonstrated in Figure 20, highlighting the hardness enhancement in mixed crystal hardening or phase separation at miscibility gaps. Notably, mixed phases of carbides with the same VEC contribute significantly to the hardness increase. These hardening effects observed in bulk materials can be, to a certain extent, transferred to layered materials, making relevant VEC elements pivotal in improving the overall properties of nitriding films [92]. The relevant VEC elements can be categorized into three groups: high VEC elements (e.g., Co, Ni), lower VEC elements (e.g., Ti, Mo, W, Nb), and nonmetallic elements (e.g., Si, C). Understanding and controlling the VEC in the film deposition process are crucial for achieving desired mechanical properties and performance in nitriding films.

Han and coworkers [195] conducted an investigation into the strengthening mechanisms of transition-metal nitride films MeNx (where Me = Ti, Zr, Hf) across a wide range of compositions at the atomic scale. They suggested that the peak properties related to the composition are not the sole reason for grain refinement, preferred orientation, residual stress, or other strengthening mechanisms. Instead, they attributed these effects to inner atomic-scale factors, specifically the changes in inner electron structures. The investigation of Han et al. supported Seung-Hoon Jhi et al. [196], who had proposed that the behavior of hardness (and correspondingly, the elastic shear modulus) of sub-stoichiometric transition-metal compounds may depend on the micro-structure of vacancies.

### 3.7. The Synergistic Mechanism

Based on the preceding discussion, we can arrive at the conclusion that the increased hardness observed in films (including nitride, carbide, and carbonitride films, as depicted in Figure 14 and Figure 19, denoting the hardness enhancement) is not solely attributed to a single strengthening mechanism. Instead, it is a multifaceted process involving various strengthening mechanisms [142]. For instance, J. Alan et al. put forward a novel theory of hardening through spinodal decomposition, highlighting the interaction between dislocations and diffused attractive obstacles. Additionally, J. Alan revealed that several relevant strengthening theories have been developed to further explain this phenomenon. In this complex process, the synergistic effect is defined as the combined influence of the following mechanisms: (1) chemical strengthening, (2) stacking fault strengthening, (3) modulus difference hardening [86,123,142,147], (4) coherency strengthening [131,136,140], (5) order strengthening. These mechanisms work together to enhance the hardness of the films in a coordinated manner.

In Section 3.1, Section 3.2, Section 3.3, Section 3.4, Section 3.5 and Section 3.6, we have introduced the strengthening mechanisms in a sequential manner: the distorted lattices effect (solid solution) [122,124,125], the grain size effect (or the Hall-Petch relationship) [38,125,133,134], the grain boundaries (internal interfaces) [131,136,137,138,139,140], the structures, and the phase formations [38,55,122,197,198], respectively. Throughout these sections, the synergy-combination relationship has been shown to play a dominant role in various strengthening mechanisms. It is crucial to emphasize that the synergistic effect is not merely a simplistic combination of these strengthening mechanisms. Instead, the enhancement of hardness is a systematic and synergistic outcome [41], with the added benefit of facilitating film lubrication to some extent. 

**Figure 21:**
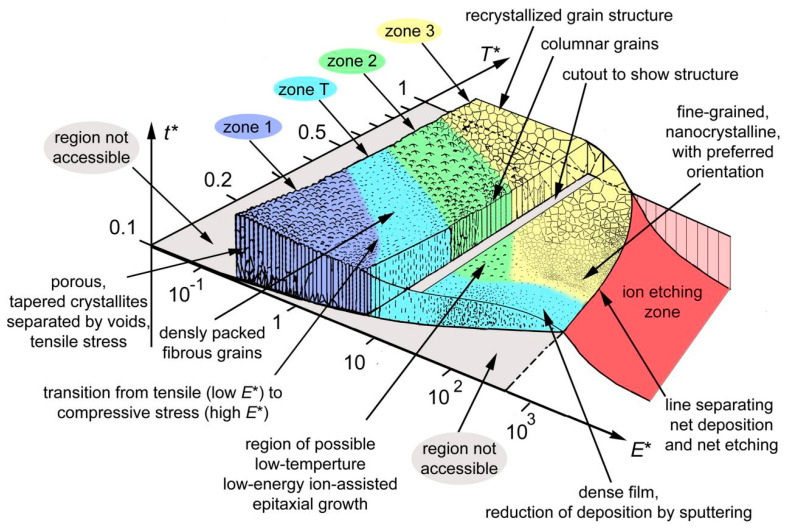
Structure zone diagram applicable to energetic deposition, adapted from ref. [199].

In Figure 21, we illustrate the synergistic mechanism using deposition parameters as an example. It is well-known that the deposition process significantly influences the properties of thin films. Recently, the energy-loading effect (Ion bombardment) has emerged as a prominent area of research for augmenting the hardness of thin films. Interestingly, the enhancement of hardness cannot be solely attributed to a single strengthening mechanism. Instead, it is achieved through an indirect synergistic mechanism by optimizing the deposition parameters [86,97,113]. During the deposition process, the bombardment of ionized particles with high energy plays a crucial role. It activates the activity of the substrate, leading to a higher nucleation density. Consequently, the grain size is controlled, and the incorporation of the amorphous phase is facilitated [3,49,50,51,52]. These interconnected processes ultimately culminate in a systematic and synergistic enhancement of hardness in the thin films. The complex interplay of deposition parameters and ion bombardment highlights the significance of considering multiple strengthening mechanisms to understand the hardness augmentation in thin films effectively. It emphasizes the importance of taking a comprehensive approach to explore and optimize deposition conditions for improved film properties.

## 4. The Lubricating Mechanism

In 1886, British scientist Osborne Reynolds provided a fundamental theory of fluid pressure lubrication based on observations made by his predecessors regarding fluid pressure phenomena. Subsequently, various theoretical frameworks were proposed: the boundary lubrication theory in 1921, the elastohydrodynamic lubrication theory in 1949, and the solid lubricant film theory in 1990. In modern times, lubrication research emphasizes two main aspects [199]: (1) reducing friction resistance and (2) slowing down wear. Lubricants play a crucial role in achieving these objectives by minimizing friction and wear between sliding contact surfaces. The scientific study of friction, wear, and lubrication is known as tribology, which is derived from the Greek words “tribos” meaning rubbing and “logos” meaning study. Tribological processes can occur in the contact area between two friction partners and can involve physical, physicochemical (e.g., adsorption, desorption), or chemical interactions (tribo-chemistry) [200]. In Section 4, we will briefly discuss the lubricating mechanisms in a temperature range from RT to elevated temperatures. This exploration will shed light on the critical factors that influence lubrication performance in different thermal conditions.

In Section 2.2.1, Figure 11 illustrates a graphical representation of the effective temperature range for different groups of solid-lubricating materials, commonly known as solid lubricants. The graph shows that no single material can fully meet all the tribological requirements across the entire temperature range, from RT to extremely high temperatures (approximately 1000 °C). To address this challenge, researchers have adopted a combination strategy that involves incorporating various solid lubricants together. This approach has proven effective in widening the lubricating temperature range, allowing lubricants to perform optimally even at temperatures close to 1000 °C [54,116,201,202,203,204]. By utilizing a mix of solid lubricant components, it becomes possible to achieve superior lubrication performance over a broader temperature range. Considering the current state of nitride coatings, most of them are composite coatings. Therefore, this discussion on lubrication mechanisms will focus on the perspective of the solid lubricant components introduced into these films. Investigating the behavior and interactions of these solid lubricant constituents within the films will provide valuable insights into the lubricating mechanisms at work.

### 4.1. The Lubricating Mechanism Based on the Nature of Constituting Material

#### 4.1.1. The Laminar Materials

**Figure 22:**
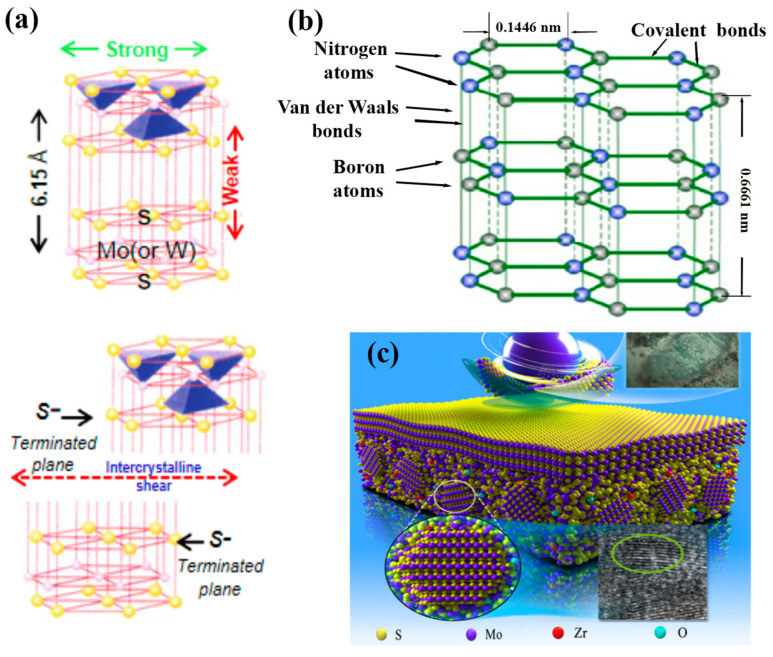
Crystal structures demonstrating the Van der Waals bonds present in the interlamellar layers/layers resulting in an easy slip between them (**a**) MoS_2_ (or WS_2_); and (**b**) hexagonal boron nitride (h-BN); (**c**) schematic diagram for illustrating the tribological behavior of film with nanostructures. (**a**,**b**) adapted from ref. [107], (**c**) adapted from ref. [32].

The laminar materials, such as graphite [205,206] and h-BN [105,106,107,207,208], as well as MoS_2_ [207,209,210], which is called the “super slippery solid” by Somuri Prasad [211], whose structure is shown in Figure 22a, shear easily due to their unique inter-layered structures and the low inter-layer bondings. In addition to MoS_2_ and graphite, laminar solids [106,212] include several sulfide, selenide, and telluride compounds, as well as lead oxide (PbO) [213,214,215].

In the context of improving tribological behavior, let us consider the example of hexagonal boron nitride (h-BN) to shed light on the significance of laminar materials. Figure 22b illustrates the layered structure of h-BN [207,216,217]. It crystallizes in a hexagonal arrangement, where BN layers are stacked on top of each other. The bonding between boron and nitrogen atoms within the layers is covalent, while the interactions between adjacent BN layers are relatively weak Van der Waals forces [218], leading to interplanar mechanical weakness. Due to this structural arrangement, when subjected to a shear force, intercrystalline slip occurs in the weak interplanar regions. As a result, during wear, smooth transfer films are formed, which can be easily sheared during relative sliding motions [218,219]. These transfer films act as an effective lubricating layer, providing lubrication and reducing friction between the sliding surfaces. The laminar structure of materials like h-BN facilitates the formation of transfer films during wear, which act as lubricating layers. This property allows for smooth and easy shearing during sliding, thereby improving the tribological performance of the material.

#### 4.1.2. The Soft Metal

**Figure 23:**
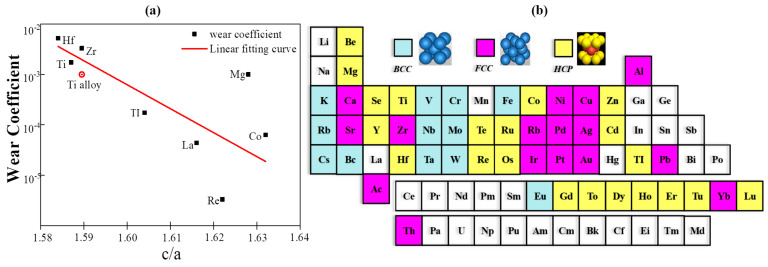
(**a**) Wear coefficient vs. c/a for HCP structured metal elements, adapted from ref. [230]; (**b**) Periodic table showing metal elements with BCC, FCC, HCP structures.

In nitride composite coating systems, soft metals [105,106,107], such as Ag [220,221,222,223,224], Cu [103,205,225], Au [106,226], Pb [227,228], etc., are usually taken as incorporations to supply lubrication at moderate temperatures. Silver (Ag), being a classic soft metal, has demonstrated its usefulness and excellence as a low-to-moderate-temperature solid lubricant in composite coatings [106]. Soft metals, such as those with a face-centered cubic (FCC) crystal structure, have the advantage of easy shear, providing favorable lubricating conditions during sliding. This property allows them to act as effective solid lubricating materials [229]. Akhtar et al. have revealed [107] that the primary reason metals act as lubricants is their low shear strength, which becomes even lower at elevated temperatures. This characteristic makes them suitable for use as solid lubricants in various applications, particularly at higher operating temperatures. Additionally, Figure 23a illustrates a relationship proposed by E. Rabinowicz and coworkers [230] between the wear coefficient and the c/a ratio for hexagonal close-packed (HCP) structured metals. The figure indicates that a higher c/a ratio in HCP metals leads to a significantly reduced wear coefficient. It has been observed that alloying metals, such as titanium, to increase the c/a ratio, results in lower wear rates.

Based on the discussions above, we can conclude that both FCC and HCP structured metals show lubrication behavior at appropriate operating temperatures with different internal mechanisms. Of all the mechanisms, the nature of low shearable bonds is the universal and basic one. The FCC, BCC, HCP structured metals are shown in Figure 23b.

### 4.2. The Lubricating Mechanism Based on the Tribo-Chemical Reactions

The incorporation of specific elements into the coating matrix can lead to a wide range of enhanced properties, making the coated materials more resilient, resistant to oxidation and corrosion, and offering improved lubricating performance and hardness. The combination of structural and compositional design, along with tribo-chemical interactions during friction, plays a crucial role in achieving the overall enhancement of the coating’s tribological behavior. According to Donnet [15], the introduction of elements such as Al, Si, B, Ag, etc. [98] into the matrix can significantly enhance a range of properties, including thermal stability [38,231], oxidation resistance [232], corrosion resistance [233], and lubricating performance [34,53,234], as well as hardness [53,123,235]. These improvements can be achieved through strategic structural design or composition design [236]. In modern practice, the films used are typically doped or composite, with the incorporated components serving as reinforcements or lubricants. Tribo-chemical reactions [237,238] take place in the contact area during friction, and some of the resulting friction products possess lubricating properties. This effect contributes to a reduction in the friction coefficient and a decrease in the wear rate.

**Figure 24:**
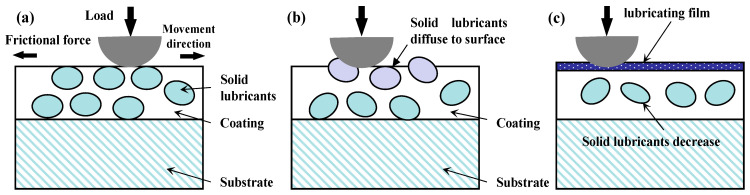
Schematic illustration of the formation of tribo-chemical reactions. (**a**) no sliding friction stage; (**b**) initial stage of friction; (**c**) formation stage of lubricating film. Adapted from ref. [239].

Figure 24 provides a vivid illustration of the formation of tribo-chemical products in three steps [239]. In Step One, the additional incorporations [71] with specific functionalities, such as reinforcements and lubricants, are embedded [3,71,76,240] into the coating. This stage does not involve sliding friction. In Step Two, as the load is applied to the coating, solid lubricants begin to migrate to the surface. This marks the initial stage of friction, during which soft metal lubricants diffuse to the film surface [201,225] due to the heat generated by friction [109,110,111]. In Step Three, a solid lubricating film, also known as the tribo-glaze coating, develops on the coating surface. The presence of solid lubricants [241,242] on the surface leads to a significant reduction in the friction coefficient. In the following discussion, we will focus on the lubricating mechanisms triggered during the tribo-chemical reactions, specifically with examples related to the formation of oxides and inorganic salts as tribo-chemical products. These reactions play a crucial role in enhancing the lubrication performance of the coating.

#### 4.2.1. The Lubricious Oxides

The rapid formation of oxides in the sliding contact area is a result of the heat generated by friction [110]. Early investigations [107,239,243] have provided insights into the influence of tribo-oxidation on friction and wear. It has been identified that oxygen plays a significant role in promoting tribo-chemical reactions at the contact surface of the coating, particularly at elevated temperatures. This effect becomes evident in the coefficient of friction and wear, which are noticeably higher in an oxygen atmosphere compared to a nitrogen atmosphere. The presence of oxygen enhances tribo-chemical processes, leading to reduced friction and wear on the coating’s contact surface. This discovery emphasizes the importance of comprehending the role of oxygen in tribo-chemical reactions and its impact on the tribological behavior of coated materials. Understanding these mechanisms is essential for optimizing the performance and durability of coated materials in various frictional environments.

(1) Oxides with low-shear-strength

Here, we will discuss the significance of low-shear oxides, using the Magnéli phase as an example to illustrate their role in facilitating tribological issues. The formation of low-shear oxides, like the Magnéli phase, through tribo-oxidation at sliding interfaces, plays a crucial role in affecting the friction and wear characteristics of materials. The slight variation in oxygen content in these oxides can lead to significant changes in shear strength, resulting in reduced friction coefficient values and improved tribological performance, especially at elevated temperatures. The Magnéli phase was first discovered by Magnéli in 1953 [244]. These substoichiometric compounds of certain transition metals constitute a homologous series with the formula Me_n_O_2n−1_, Me_n_O_3n−1_, or Me_n_O_3n−2_, known as the magnéli phases [245]. Examples of these phases include TiO_x_, VO_x_, and MoO_x_. Here we will take TiO_x_ as an example to decipher the simple (low-shear-strength) but significant tribological mechanism. Gardos et al. [246] argued that a slight decrease in oxygen content from stoichiometric TiO_2_ to under-stoichiometric TiO_x_ (where x is in the range of 1.93 to 1.98) resulted in a decrease in the shear strength from 21 MPa to 8 MPa. This decrease in shear strength led to very low friction coefficient values of around 0.08 for x = 1.98. Furthermore, Woydt et al. [247,248] proposed that the Magnéli phases of the Ti_n_O_2n−1_ type (γ-Ti_3_O_5_, Ti_5_O_9_, and Ti_9_O_17_) are also beneficial in reducing sliding friction and wear at elevated temperatures.

Along with the development of theoretical research, Thomas et al. [249] employed DFT to calculate the distortion of V_2_O_5_, and they found that the screened Coulomb potential between layers leads to lower elastic constants and the octahedral structure results in large layer distances, leading to the presence of classic Magnéli phase V_2_O_5_ with low shear strength.

(2) Oxides with low melting points

Heat is generated when sliding occurs between counterparts, leading to an increase in the temperature of the contact area [109,110,111]. Usui et al. [250] reported that the highest temperature at the contact area can rise to almost 500 °C during sliding at ambient temperature. Additionally, certain metal additives may diffuse to the film surface and subsequently undergo oxidation, resulting in the formation of metallic oxides. 

Oxides with low melting points can form a liquid phase (or liquid-like phase) during the dry friction process. When the relative sliding areas are covered by the liquid-phased oxide, the tribological model transitions from solid film lubrication to liquid (or quasi-liquid/liquid-like) lubrication [251,252]. Early studies [213,214,215] have reported that lead oxide (PbO) demonstrates effective solid oxide lubrication, exhibiting a friction coefficient of 0.07 at 675 °C. The good lubricity is attributed to the viscous flows of soft PbO. The tribological properties of vanadium oxide in relation to temperature were investigated by Gulbinski et al. [253] and Fateh et al. [254], respectively. Gulbinski et al. [253] observed that the coefficient of friction (COF) decreased from 0.8 to 0.3 when the films were tested in a temperature range from 100 °C to 700 °C. This trend agrees with the melting point (T_m_) of V_2_O_5_, which is 690 °C. The COF values reported by Fateh et al. [254,255] were lower in a similar temperature range (from 0.55 to 0.15 when tribotested from RT to 600 °C) but displayed a similar trend.

In summary, we can conclude that the melting-induced effect has been confirmed, indicating that the coefficient of friction (COF) is lower for liquid-like (or quasi-liquid) materials. This phenomenon can be primarily attributed to the transition from solid film lubrication to liquid (or quasi-liquid) lubrication, as proposed in the tribological model [256,257].

(3) Oxides with high-ionic potential

**Figure 25:**
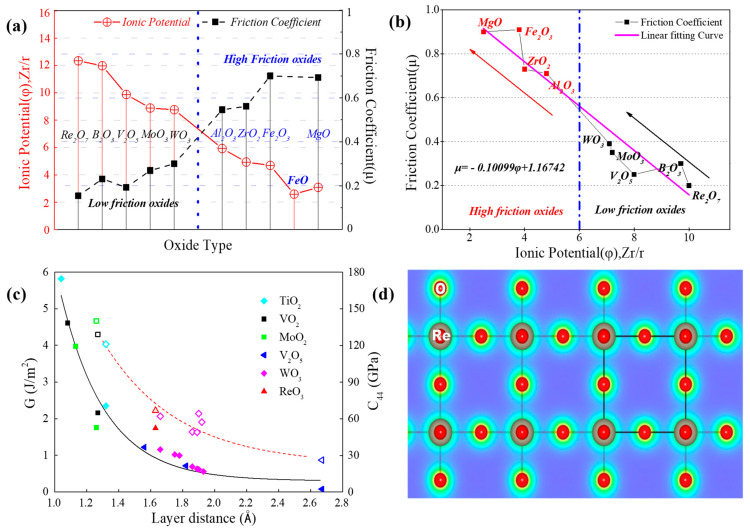
Relation between ionic potentials (φ) and averaged friction coefficients (μ) of various oxides, (**a**) ionic potential is defined as φ = Z/r (Z is the cationic charge, r is the radius of the cation); (**b**) the linear fitting curve of the oxides, (**a**,**b**) adapted from ref. [259]; (**c**) decohesion energy G (filled circles) and elastic constant C44 (open circles) as a function of the original distance d between the cleaved layers; (**d**) structure of ReO_3_ projected along the [100] axis, (**c**,**d**) adapted from ref. [226].

Ali Erdemir et al. [258,259,260] introduced a crystal-chemical approach known as the Ionic Potential Model [255] which has been employed to study various nano-composite coatings [106,261,262]. In general, oxides with higher ionic potentials tend to exhibit lower friction coefficients at elevated temperatures. On the other hand, oxides with lower ionic potentials, such as Al_2_O_3_, Fe_2_O_3_, and MgO, possess considerable strength, making them resistant to shear. The fundamental basis of this crystal-chemical model lies in the ionic potential (u) of an oxide, as illustrated in Figure 25a,b (Adapted from [259]). A higher ionic potential results in enhanced screening of a cation within an oxide by surrounding anions. Oxides with highly screened cations, for example, V_2_O_5_, WO_3_, and Re_2_O_7_, tend to be soft and consequently exhibit easy shear, leading to lower friction at elevated temperatures. Their cations are separated well and effectively screened by oxygen anions, limiting their capacity to undergo extensive chemical interactions with other cations due to the majority of their bonding sites being surrounded by oxygen anions. Moreover, in the case of binary oxides, the crystal-chemical approach considers the difference in ionic potentials between the two oxides present on sliding surfaces. Under such circumstances, a larger difference in ionic potentials results in lower friction coefficients [259,262].

In addition to the previously mentioned oxides with characteristics like (1) low shear strength, (2) low melt point, and (3) high ionic potential, it is likely that these three groups share some common candidates with interconnections yet to be discovered. Thomas Reeswinkel et al. [263] investigated the relationship between coulomb-potential and shear strength for Magnéli phases, as depicted in Figure 25c. On the other hand, Prasad et al. [264] and Zabinski et al. [265] reported on ZnO, a particularly noteworthy non-Magnéli phase, where solid lubrication is attributed to a defective nano-columnar grain structure (sub-grains) containing a high density of (0002) basal plane ZnO stacking faults. ZnO coatings with such a defective structure exhibit room temperature friction coefficient values in the range of 0.1 to 0.2, along with wear rates around 1 × 10^−7^ mm^3^/(N·m). This favorable structure allows for plastic deformation and ductility, resulting in low shear properties.

#### 4.2.2. The Lubricious Inorganic Salts

**Figure 26:**
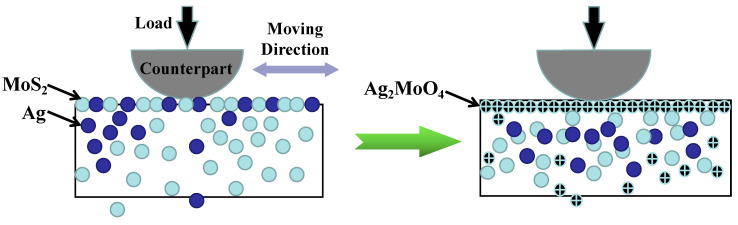
The formation of inorganic salts Ag_2_MoO_4_.

The lubricating mechanisms of inorganic salts are intricate, and they can differ significantly among various types of inorganic salts. Rather than being attributed solely to a single mechanism, the lubricating effects of these salts often arise from a synergistic combination of factors [15,266,267,268]. In this context, we will examine the lubricating mechanisms of two specific inorganic salts, namely Ag_2_MoO_4_ [266] and AgTaO_3_ (recently demonstrated to exhibit an extremely low coefficient of friction (COF) of 0.06 at 750 °C) [202,267]. Figure 26 illustrates the process of forming the inorganic salt Ag_2_MoO_4_ (also referred to as tribo-glaze), wherein the lubricating components of Ag and MoS_2_ can be observed. During friction, the soft metal Ag diffuses to the surface, where it subsequently reacts with MoS_2_ in an oxygenated atmosphere, triggered by the heat generated due to friction. This reaction leads to the formation of the tribo-glaze, Ag_2_MoO_4_ [109,110,111]. Ag_2_MoO_4_ has a melting point of 483 °C, which causes the transition from dry friction to boundary (or quasi-liquid) lubrication at elevated temperatures above 483 °C. The crystal structure of a silver molybdate, such as Ag_2_MoO_4_, can be described as comprising mixed Ag_2_O and MoO_3_ layers bonded together by silver ions [266]. These layers feature easily breakable Ag-O and O-Ag-O interlayer bonds, which have been proposed as the contributing factor to the observed high-temperature solid lubricity. Furthermore, the ionic potential between Ag_2_O and MoO_3_ may also offer an additional explanation for this phenomenon [259,262].

S. Stone et al. [202] have established that AgTaO_3_ possesses a classic binary metal oxide structure with distinct layers. As temperature increases, AgTaO_3_ undergoes a series of structural phase transitions until reaching its melting point (1172 °C). The dry sliding wear tests of the coatings against Si_3_N_4_ counterfaces have revealed friction coefficients in the range of 0.06 to 0.15 at temperatures ranging from room temperature to 750 °C. These findings strongly indicate that AgTaO_3_ serves as an exceptional high-temperature solid lubricant. Aouadi et al. [201,202,226], on the other hand, propose that compounds like Ag_2_MoO_4_ and Ag_2_Mo_2_O_7_ exhibit lubrication mechanisms due to layer sliding during relative motion caused by weak chemical bonds. Consequently, the primary mechanisms driving the lubricious properties of inorganic salts are their low melting points [266] and layered structures [106].

Table 1 presents the coefficient of friction (COF) for several other inorganic salts (binary metal oxides). It is evident from the data that these salts exhibit relatively low COF values at testing temperatures above 700 °C compared to other high-temperature solid lubricants. This characteristic further supports the notion that inorganic salts, especially binary metal oxides, hold significant promise as effective high-temperature solid lubricants.

### 4.3. The Lubricious Fluorides

Fluorides of alkaline-earth metals, e.g., CaF_2_, BaF_2_, and CeF_3_ [269,270,271,272], as well as the famous PS [60,61,62,63,64,65,66,67] homologous coatings developed by NASA, which contain fluorides as efficient high-temperature solid lubricants, have demonstrated remarkable lubricating properties within the temperature range of 500 °C to 900 °C. While most fluoride compounds tend to be brittle and non-lubricating at temperatures below 500 °C, they undergo a transition to the plastic state above 500 °C, becoming effective lubricants. This transition is attributed to the low shear strength of chemical bonding after reaching the plastic state [106,273]. To satisfy the continuous lubrication requirements at elevated temperatures, these lubricious fluorides are typically incorporated into the coatings as lubricants. For example, the lubricious coatings of the PS series, as discussed in Section 2.1.1, achieve their high-temperature lubricating performance by including fluoride additions of BaF_2_ and CaF_2_ (10 wt.% together) [68]. This approach ensures that the coatings maintain their lubricating properties even under demanding high-temperature conditions [68].

### 4.4. The Lubricating Mechanism Based on Textures

**Figure 27:**
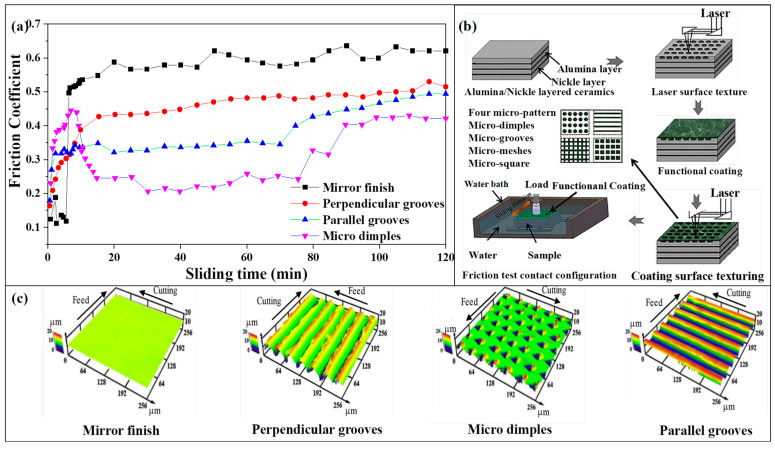
Schematic diagrams of laser surface texturing workpiece, adapted from ref. [282] for (**b**), ref. [284] for (**a**,**c**).

Previous investigations [274,275,276,277,278,279,280,281] have highlighted the successful integration of solid lubricant films with smart surface engineering strategies, such as micro-texturing and/or micro-patterning, leading to even higher levels of performance and durability under severe tribological conditions. Notably, Fan and colleagues [282] have introduced an innovative design strategy for fabricating a double-layered texture, which has proven highly effective in achieving the desired lubricating objectives of low friction coefficient and high wear resistance. One specific example to illustrate the lubricating mechanisms of surface micro-texturing is the use of micro-dimple patterns. Through the application of micro-patterns on the coating, the dimples serve as reservoirs capable of storing lubricants, both solid and/or liquid, which can be released during sliding to reduce friction and wear. Additionally, these dimples temporarily retain wear debris, thereby slowing down adhesive wear. Importantly, it is evident that this surface-textured approach can be applied to various types of film [283]. Figure 27 provides a clear and vivid schematic representation of the laser surface texturing processing involved in this technique.

### 4.5. The Lubricating Mechanism Based on the Synergistic Effect

**Figure 28:**
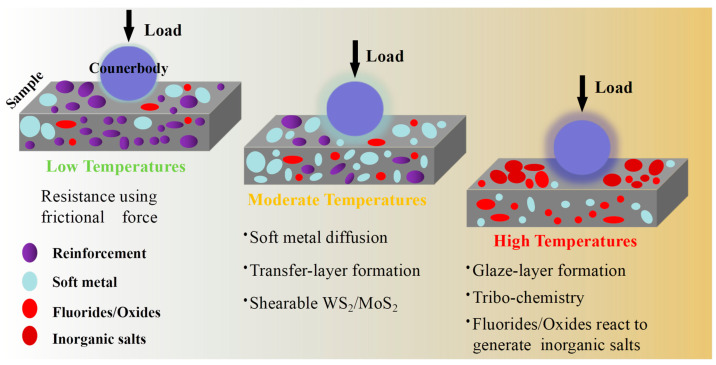
Schematic diagrams showing synergism of solid lubricants, i.e., soft metal, laminar solids, and fluorides to broaden the range of lubricating temperature, adapted from ref. [105].

Figure 28 demonstrates the synergistic lubricating mechanisms [68,282] involved in solid film lubrication, and it is evident that these mechanisms vary across different temperature ranges. At low temperatures, the dominant lubricating mechanism is the reduction of friction force. As the temperature increases to moderate levels, the lubrication mechanisms involve lubricant diffusion, utilization of soft metals (e.g., Ag [54,220,221,222,223,224], Cu [103,205,225], Au [106,226], Pb [227,228]), use of easy-shear materials such as graphite [3,205,206] and h-BN [105,106,107,207,208], as well as two-dimensional layered metal sulfides [207,209,210] (WS_2_ and/or MoS_2_) and/or Magnéli oxides [244,245,246,247,248,249] (e.g., Re_2_O_7_, V_2_O_5_, WO_3_, MoO_3_, B_2_O_3_, etc.), and the formation of transfer-layers [15,201,202,226,259,262,266,267,268]. As the temperature rises to high levels, the lubricating performance further improves due to the formation of a glaze layer [212] and the involvement of inorganic salts and tribo-chemical reactions. These lubricating mechanisms synergistically operate over a wide temperature range, ensuring continuous lubrication [276]. This comprehensive understanding of the lubricating mechanisms is essential for optimizing solid film lubrication performance under various operating conditions.

## 5. Outlooks

In modern industry, the demand for solid lubrication materials with exceptional hardness and toughness, while maintaining a low coefficient of friction (less than ~0.2) and a low wear rate (less than 10^−8^ mm^3^/(Nm)), presents a significant challenge. This challenge becomes even more pronounced when considering a wide temperature range, from room temperature (RT) to high temperatures. Although considerable progress has been made in investigating mechanisms for strengthening, toughening, and lubricating materials, the field of film tribology at high temperatures still lacks a systematic and fundamental theory, primarily relying on the experience of solid lubrication theory. To address this, further studies are required to achieve significant advancements in reducing wear and friction of solid lubricating materials at high temperatures. Particularly, there is a growing need to pursue zero or near-zero wear in many MEMS, NEMS, small-scale devices, and precision mechanical systems. Such advancements would have far-reaching implications for various industrial applications and help meet the ever-increasing demands of modern technology.

### 5.1. Refine the Existing Theory

The theory of solid film lubrication was initially proposed in the 1990s, and substantial advancements have been achieved in elucidating film lubrication phenomena. Nevertheless, the absence of a unified theory hinders a comprehensive understanding of the synergistic effect of strengthening and toughening. Additionally, a lack of a unified mathematical model to describe the relationship between tribological behavior and hardness presents a challenge. Furthermore, the absence of a guiding theory for the development of a single lubricant capable of meeting lubrication requirements across a wide temperature range, from room temperature (RT) to high temperatures, is another prevailing issue. To address these deficiencies, it is imperative to enhance and refine the existing theory of solid film lubrication. By addressing these shortcomings, researchers can pave the way for designing and developing advanced lubrication materials that perform exceptionally well across diverse temperature ranges. An improved and comprehensive theory would not only provide valuable insights into strengthening and toughening mechanisms but also facilitate the creation of superior lubricants to fulfill lubrication expectations effectively.

### 5.2. Application of Data-Driven Research

With the passage of time, material research has evolved through three distinct stages: empirical science, theoretical science, and computer simulation. In the present era, data-driven research is increasingly gaining prominence, playing a vital role in the development of various novel materials. Notably, significant progress has been made in the high-throughput preparation and characterization of nitride films by leveraging databases. This shift marks a progressive transition in material research methods from knowledge-driven approaches to data-driven methodologies. The utilization of data-driven research approaches has proven to be instrumental in accelerating the discovery and development of new materials. By harnessing the power of data and advanced computational techniques, researchers can efficiently explore vast material databases, leading to the identification of promising candidates and properties. This shift towards data-driven methodologies has opened exciting possibilities for innovations in materials science and engineering, revolutionizing the way we discover, design, and optimize materials for various applications.

### 5.3. New Strategy in Guiding Design

The design strategy presented in this article represents a comprehensive approach; however, despite its strengths, it has not yet resulted in the creation of a simple and single-component coating possessing both outstanding hardness and lubrication properties. Therefore, there is a need for a novel and alternative strategy that can guide the design of films with new components and/or structures, enabling the development of coatings that exhibit satisfactory hardness and lubrication performance across a wide temperature range and diverse environments. To achieve this, researchers should explore innovative approaches that combine multiple materials or structures, capitalizing on synergistic effects to enhance both hardness and lubrication capabilities. Additionally, the incorporation of advanced nanotechnology, surface engineering techniques, and data-driven approaches can aid in the rational design of materials tailored to meet specific lubrication requirements. Such advancements would have significant implications for a wide range of industries, including manufacturing, transportation, and energy, contributing to more efficient and sustainable technologies.

## Figures and Tables

**Table 1:** Friction coefficient of sectional inorganic salts (Data from ref. [212]).

Lubricants	Test Temp. (°C)	Load (kg)	Velocity (cm·s^−1^)	Coefficient of Friction (μ)
PbMoO_4_	>700	7.7	0.76	0.29
K_2_MoO_4_				0.20
NiMoO_4_				0.29
Ag_2_MoO_4_				0.28
FeMoO_4_				0.42
Na_2_WO_4_				0.17
Pb_2_WO_4_				0.35
CuWO_4_				0.41
FeWO_4_				0.43
CaWO_4_				0.45

## Data Availability

The data presented in this review come from the corresponding references.

## Abbreviations

PVD	Physical Vapor Deposition
CVD	Chemical Vapor Deposition
RT	Room Temperature
VEC	The valence electron concentration effect
TMN	Transition metal nitride
2D	Two-dimensional
3D	Three-dimensional
Eq	Equation
Ref	Reference
nc	Nanocrystalline
α	Amorphous
AIMD	Ab initio molecular dynamics
DFT	Density Functional Theory
MD	Molecular Dynamics
TEM	Transmission electron microscope
ML	Monolayer
H	Hardness
c	Crystalline
T_a_	Annealing temperature
FCC	Face centered cubic
BCC	Body centered cubic
HCP	Hexagonal closed pack
HT	High temperature
MEMS	Micro Electro Mechanical Systems
NEMS	Nano-Electromechanical Systems
MMCs	Metal matrix composites
